# RNA stores tau reversibly in complex coacervates

**DOI:** 10.1371/journal.pbio.2002183

**Published:** 2017-07-06

**Authors:** Xuemei Zhang, Yanxian Lin, Neil A. Eschmann, Hongjun Zhou, Jennifer N. Rauch, Israel Hernandez, Elmer Guzman, Kenneth S. Kosik, Songi Han

**Affiliations:** 1 Molecular, Cell and Developmental Biology, University of California Santa Barbara, Santa Barbara, California, United States of America; 2 Neuroscience Research Institute, University of California Santa Barbara, Santa Barbara, California, United States of America; 3 Biomolecular Science and Engineering, University of California Santa Barbara, Santa Barbara, California, United States of America; 4 Department of Chemistry and Biochemistry, University of California Santa Barbara, Santa Barbara, California, United States of America; 5 Department of Chemical Engineering, University of California Santa Barbara, Santa Barbara, California, United States of America; University College London, United Kingdom of Great Britain and Northern Ireland

## Abstract

Nonmembrane-bound organelles that behave like liquid droplets are widespread among eukaryotic cells. Their dysregulation appears to be a critical step in several neurodegenerative conditions. Here, we report that tau protein, the primary constituent of Alzheimer neurofibrillary tangles, can form liquid droplets and therefore has the necessary biophysical properties to undergo liquid-liquid phase separation (LLPS) in cells. Consonant with the factors that induce LLPS, tau is an intrinsically disordered protein that complexes with RNA to form droplets. Uniquely, the pool of RNAs to which tau binds in living cells are tRNAs. This phase state of tau is held in an approximately 1:1 charge balance across the protein and the nucleic acid constituents, and can thus be maximal at different RNA:tau mass ratios, depending on the biopolymer constituents involved. This feature is characteristic of complex coacervation. We furthermore show that the LLPS process is directly and sensitively tuned by salt concentration and temperature, implying it is modulated by both electrostatic interactions between the involved protein and nucleic acid constituents, as well as net changes in entropy. Despite the high protein concentration within the complex coacervate phase, tau is locally freely tumbling and capable of diffusing through the droplet interior. In fact, tau in the condensed phase state does not reveal any immediate changes in local protein packing, local conformations and local protein dynamics from that of tau in the dilute solution state. In contrast, the population of aggregation-prone tau as induced by the complexation with heparin is accompanied by large changes in local tau conformations and irreversible aggregation. However, prolonged residency within the droplet state eventually results in the emergence of detectable β-sheet structures according to thioflavin-T assay. These findings suggest that the droplet state can incubate tau and predispose the protein toward the formation of insoluble fibrils.

## Author summary

Tau is a common neuronal protein that, under circumstances and conditions not well understood to date, self-assembles into intracellular aggregates in several neurodegenerative diseases including Alzheimer disease. These aggregates are formed of fibrous polymers. The mechanism by which this critical transition from a soluble protein to insoluble fibrous material occurs is unknown. We have discovered a novel state in which many tau molecules become compacted into a protein-rich droplet while maintaining their solubility and native-like protein conformations. Chemists refer to this dense liquid droplet state as a complex coacervate phase, and it is held together by the opposite charges of their constituents, ions, and water. In the case of the tau protein, the oppositely charged constituent is RNA. Indeed, we found that in human neuronal cell culture, tau selectively binds to a category of RNA known as tRNA. Interestingly, tau and RNA favorably condense to a complex coacervate phase when the charges between them are matched and at elevated temperatures, such that tau-RNA droplets could be observed at physiologically viable protein concentrations simply by increasing the temperature from room to physiological temperatures. When the tau-RNA–dense droplets are incubated together over time, tau transitions to a conformation similar to that found in pathological fibers. Our experiments therefore demonstrate physicochemical properties of tau that may predispose it to undergo changes associated with neurodegenerative disease.

## Introduction

Inclusions consisting of the tau protein occur in many neurological conditions with Alzheimer disease the most prominent among them. Normally, tau is in a dynamic equilibrium between a microtubule-bound and free state. Under disease conditions tau self-assembles into fibrils that eventually lead to highly insoluble polymeric inclusions known as neurofibrillary tangles. The underlying biophysical basis for the transition of tau from a microtubule-associated protein to an insoluble fibril is unknown. However, a clue comes from the observation that polyanions, such as heparin, promote tau fibrillization [1]. Although less effectively, RNA can also induce tau fibrillization [2, 3], and unlike heparin, RNA is present intracellularly, making it accessible to interact with tau.

Our experiments began with the finding that tau can bind RNA in living cells. Interestingly tau-RNA binding showed selectivity for tRNAs. This observation along with the known categorization of tau as intrinsically disordered and its ability to spread from cell to cell in a manner that resembles prions [4, 5] suggested that tau might share additional properties with other RNA-binding proteins involved in neurodegeneration. These proteins include FUS [6–8], TDP-43 [9], C9ORF72 [10, 11], hnRNPA2B1, and hnRNPA1 [12–14], all of which can undergo liquid-liquid phase separation (LLPS) from the surrounding aqueous medium into droplets in vitro. These highly protein-dense structures, also known in the literature as complex coacervates [15, 16], establish a separated liquid phase typically associated with (1) exceptionally high protein concentration [17]; (2) tunability with salt concentration and temperature [18]; and (3) multivalent electrostatic interactions involving polyelectrolytes, including RNA, single-stranded DNA and intrinsically disordered proteins (IDPs) [19]. A consensus property of a complex coacervate fluid is low interfacial tension that promotes fusion and coating, and is associated with low cohesive energy between hydrated polyelectrolyte complexes and weakly bound water constituents, consistent with high internal fluid dynamics [16, 20]. Complex coacervate chemistry has been implicated in bioinspired coating, wet adhesion, and engulfment [21–23]. RNA-based coacervates are an important organizing principle for biomolecular condensates in cell biology [17, 19, 24–26].

Here, we show that tau-RNA complexation can lead to complex coacervation. When multiple tau molecules weakly bind RNA, and overall charge matching is achieved between the polycation, tau, and polyanion, RNA, tau undergoes reversible condensation and LLPS into micrometer-sized droplets. Remarkably, within this LLPS state, tau maintains a high internal segment mobility and a locally compact conformation that protects the core region of tau known as PHF6(*), as found in dilute solution state, despite the molecular crowding associated with coacervation. In contrast, this region experiences full extension that exposes the PHF6(*) region to the solvent and stacks into β-sheets in the presence of a different polyanion, heparin, that induces irreversible fibrillization of tau [27]. The spontaneous and reversible droplet formation suggests that tau is held in a low energy-barrier fluid state between dilute solution and complex coacervate condensate, with the free energy difference toggled by interactions mediated by ions and hydration water. In fact, a systematic study of complex coacervation as a function of temperature verified the process to be entropy-driven that is likely toggled by the release of counterions and/or hydration water that reduces the net excluded volume of the hydrated biopolymer constituents. However, prolonged residence in this phase state begins to induce β-sheet formation, suggesting that the highly condensed phase state of tau can be a precursor to fibril formation.

## Results

### Tau binds RNA in living cells

**Fig 1:**
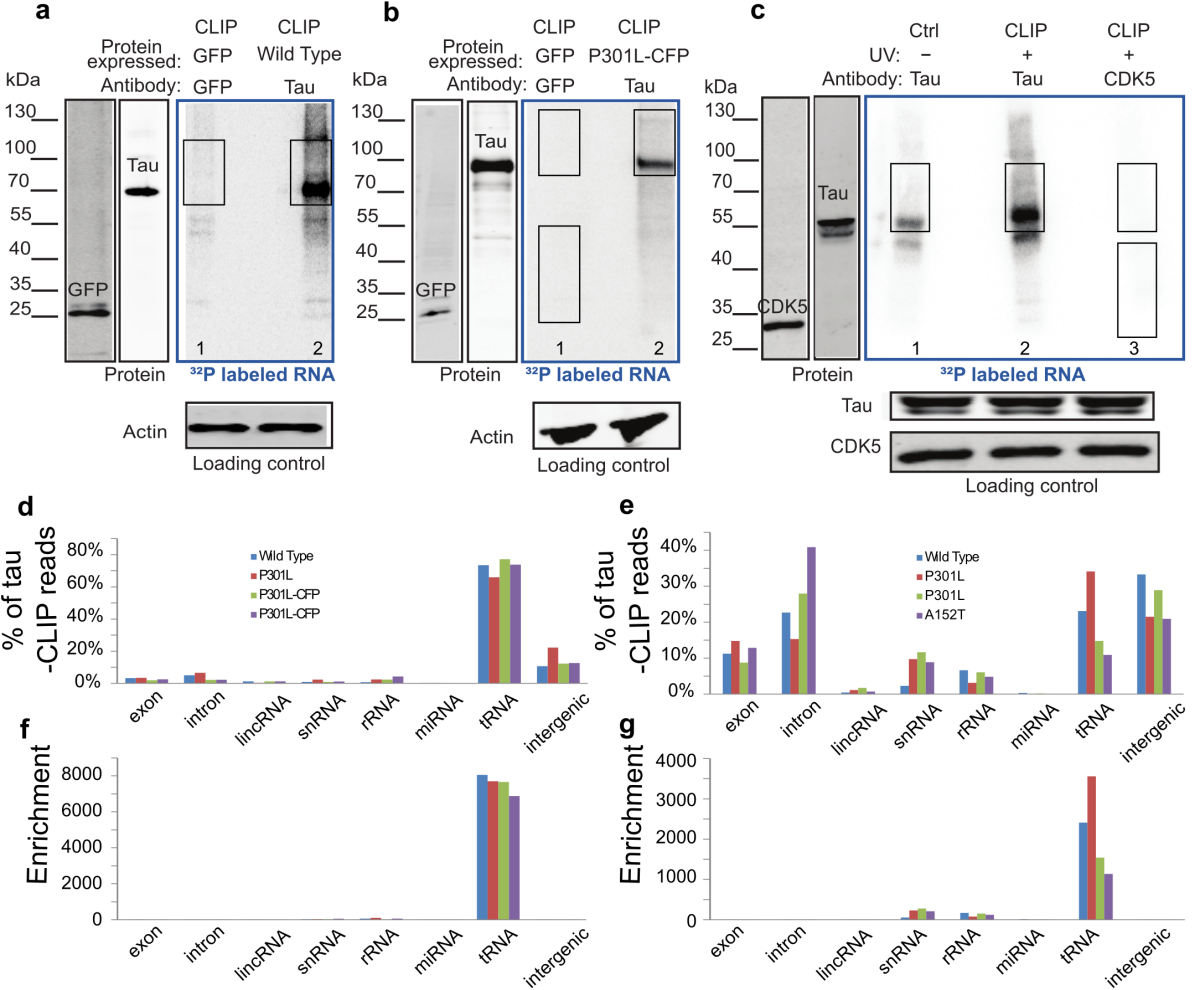
Tau PhotoActivatable Ribonucleoside-enhanced Individual-nucleotide resolution UV Cross-Linking ImmunoPrecipitation and high-throughput sequencing (PAR-iCLIP) in tau-expressing human embryonic kidney cells (HEK) and human-induced pluripotent stem cell (hiPSC) -derived neurons. Phosphor images in the blue frames (A-C) show ^32^P-labelled RNA crosslinked to tau protein in HEK cells expressing tau (A-B) and in hiPSC-derived neurons with endogenous tau (C). PAR-iCLIP in HEK cells expressing wild-type tau (A, lane 2) or tau P301L-CFP (B, lane 2; nota bene: the fused cyan fluorescent protein [CFP] retards the migration of tau). (C) PAR-iCLIP of endogenous wild-type tau in hiPSC-derived neurons (C, lane 2). The antibodies anti-tau HJ 8.5, anti-green fluorescence protein (GFP) and anti- Cyclin–dependent kinase 5 (CDK5) were used for protein precipitation. No RNase was added, unless specified. PAR-iCLIP with GFP as a control (A-B, lane 1), with CDK5 as a control (C, lane 3) and no UV control (C, lane 1). A small RNA signal was visible in the absence of cross-linking (no UV control) in hiPSC-derived neurons (C, lane 1), suggesting a small portion of RNA may associate with tau in vitro after cell lysis. The RNA-protein complexes from Cross-Linking ImmunoPrecipitation (CLIP) marked by rectangles were cut from the blot for DNA library preparation. Note that 2 regions of GFP and CDK5 were cut out as sequencing controls in which the lower molecular weight (MW) band corresponds to GFP or CDK5. (D-E) Percentage of tau-CLIP reads that are mapped to 8 human genome regions in HEK cells (D) and hiPSC-derived neurons (E). (F-G) Enrichment of tRNA in tau-CLIP of HEK cells (F) and hiPSC-derived neurons (G) as discussed in text. The numerical data used in (D-G) are included in S1 Data.

RNA binding to tau in living cells was examined by PhotoActivatable Ribonucleoside-enhanced Individual-nucleotide resolution UV Cross-Linking ImmunoPrecipitation and high-throughput sequencing (PAR-iCLIP) using the human tau-specific antibody, HJ 8.5. The tau constructs and tau mutants used in this study are shown in S1 Fig. Human embryonic kidney (HEK) 293T cells expressing wild-type, full-length human tau (4R2N), mutant tau (P301L-4R2N), or mutant tau in a different isoform fused to cyan fluorescent protein (CFP; P301L-4R1N) were cross-linked, and tau immunoprecipitated with the tau antibody (Fig 1A and 1B and S2A–S2C Fig). Wild-type, human-induced pluripotent stem cell (hiPSC)-derived neurons (Fig 1C), as well as lines harboring P301L tau and a risk variant for progressive supranuclear palsy, A152T were cross-linked and immunoprecipitated. A retinoic acid-differentiated neuroblastoma line (SH-SY5Y) that expresses both tau and the short isoform of MAP2, called MAP2c [28], was also cross-linked and immunoprecipitated (S2D Fig). ^32^P-labeled RNA bands correspond to immunoprecipitated cross-linked tau-RNA complexes (Fig 1A–1C, lanes 2; S2A Fig, lane 2; S2D Fig, lane 2), with strong binding of tau to RNA was observed in all these experiments. Tau-RNA complexes were observed regardless of the tau genotype. PAR-iCLIP experiments with varying RNase concentrations did not shift the ^32^P-labeled band, nor change its intensity (S2B Fig, lanes 2–3). The radioactive RNA band ran close to the tau protein itself, in contrast to that of most known RNA-binding proteins in the literature [29, 30] that run at a range of higher molecular weights. This radioactive tau band was cut from the membrane, and when sized on an RNA gel, ran in the range of 30 to 100 nucleotides. This confirms the presence of RNA, and suggests that tau bound predominantly to small RNAs or RNA fragments. Selectivity was further confirmed by showing that MAP2 did not bind RNA despite its proline rich and microtubule binding domains that are highly homologous to that of tau (S2D Fig, lane 3).

Because small RNAs are abundant and may engage in nonspecific interactions, we performed multiple confirmatory controls, including immunoprecipitation in the absence of tau expression, the use of non-tau antibodies rather than HJ 8.5 to rule out nonspecific binding, and without UV exposure to eliminate the possibility that tau-RNA complexes formed in vitro after cell lysis (Fig 1A and 1B, lane 1; Fig 1C, lanes 1 and 3; S2B Fig lanes 1 and 4; S2C Fig, lanes 3 and 4; S2D Fig, lanes 1, 3, and 4).

To identify the types of RNA crosslinked to tau, DNA libraries were prepared from the immunoprecipitated radiolabeled bands and sequenced. We analyzed the distribution of tau-bound RNA from the human genome by defining 8 regions: exons, introns, long intergenic noncoding RNAs (lincRNAs), small nuclear RNAs (snRNAs), rRNAs, microRNAs (miRNAs), tRNAs, and intergenics. In tau-expressing HEK cells, tRNAs were overwhelmingly the highest category of RNA crosslinked to tau (Fig 1D). Endogenous tau in hiPSC-derived neurons also bound tRNAs; however, background RNA from introns and intergenics were relatively abundant as well (Fig 1E). Background RNA sequences are common in all Cross-Linking ImmunoPrecipitation (CLIP) studies, particularly for atypical RNA binding proteins [31]. Despite the abundance of tRNAs in cells, however, background reads from CLIP experiments consistently show a paucity of tRNAs [29, 32–35]. Correcting for background reads by dividing the percentage of tau-bound RNA by the percentage of the nucleotides of each category in the genome demonstrated a selective enrichment of tRNAs that bind to tau in both HEK cells (Fig 1F) and hiPSC-derived neurons (Fig 1G). Furthermore, in all experiments, the non-tau controls consistently showed relatively few total reads and very few uniquely aligned reads (S2E and S2F Fig).

The specific tRNA species crosslinked to tau overlapped extensively between the HEK and hiPSC-derived neuron samples. Of 625 annotated tRNA loci in the human genome, 462 of the tRNA genes crosslinked to tau in HEK cells, with 79% of them observed in all 4 tau CLIP samples and 94% in at least 2 samples. In the hiPSC-derived neurons, all 231 tRNA genes identified were also observed in the HEK cells and 119 of these were verified in at least 2 tau CLIP samples. The distribution of the tRNAs cross-linked to tau in HEK and hiPSC-derived neurons differed markedly from the total endogenous tRNA distribution. This difference indicated that the tRNAs selected by the CLIP experiment were nonrandomly drawn from the total tRNA pool (S3A Fig and S3B Fig). Among the most differentially selected tRNAs by CLIP was tRNA^Arg^ (S3C Fig).

**Fig 2:**
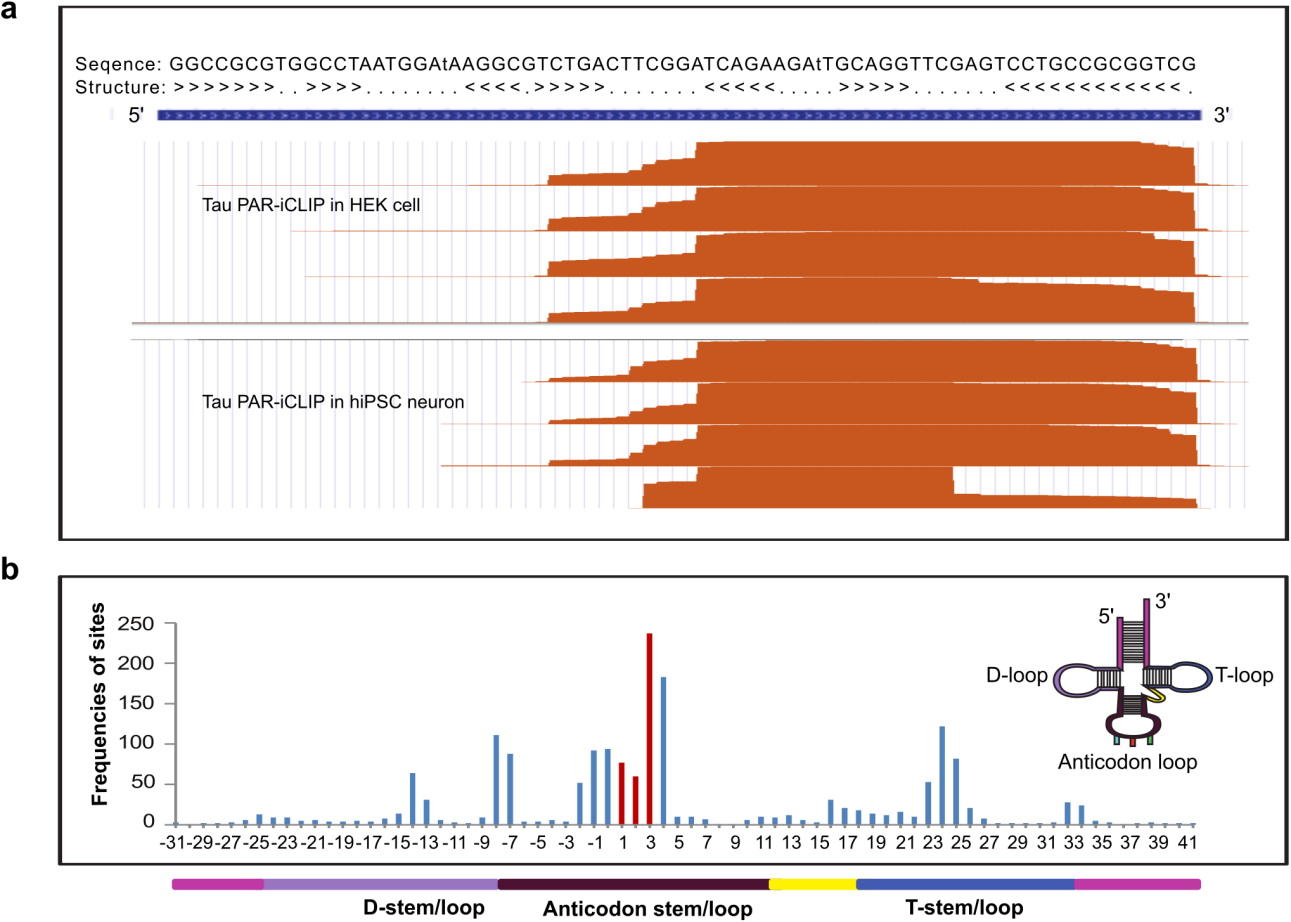
Enrichment of tRNA in PhotoActivatable Ribonucleoside-enhanced Individual-nucleotide resolution UV Cross-Linking ImmunoPrecipitation and high-throughput sequencing (PAR-iCLIP) data. (A) Cross-Linking ImmunoPrecipitation (CLIP) cDNA reads from tau expressed in human-induced pluripotent stem cell (hiPSC)-derived neurons (neuron CLIP) and from tau expressed in human embryonic kidney (HEK) cells (HEK cell CLIP) that aligned to the chr15.tRNA4-ArgTCG tRNA were found in all CLIP samples, and demonstrated a similar pattern of crosslinking. (B) Analysis of cross-linked sites along tRNA secondary structure demonstrates the anticodon preference, in which the anticodon (colored red) region is designated as position 1–3 for alignment purpose. The colored illustration of tRNA secondary structure is displayed as an inset, and below the *x* axis in one dimension. The numerical data used in (B) are included in S1 Data.

PAR-iCLIP identifies the cross-linked sites in the tRNAs (Fig 2A and 2B). We found the tRNA sequences extend from the most 3ʹ nucleotide of the tRNA to the covalently cross-linked site where the sequencing terminates (S4 Fig). In both HEK cells and hiPSC-derived neurons, the cross-link site was predominantly located within the anticodon loop followed by the T-loop and in the D-loop, but the latter 2 with far smaller frequencies (Fig 2B). The observed preference for single-stranded segments of tRNA as cross-link sites to tau, is likely due to the crosslinking between the nucleobases in this region and aromatic rings on the protein. We conclude that tau selectively binds RNA, and the predominant RNA specie bound is tRNA. CLIP procedures are not highly quantitative, and therefore cannot resolve differences in tau-RNA binding among the tau variants tested, P301L, or A152T. Therefore, we are unable to conclude whether tau mutations affect its RNA binding.

### Tau-RNA binding affinity and stoichiometry

**Fig 3:**
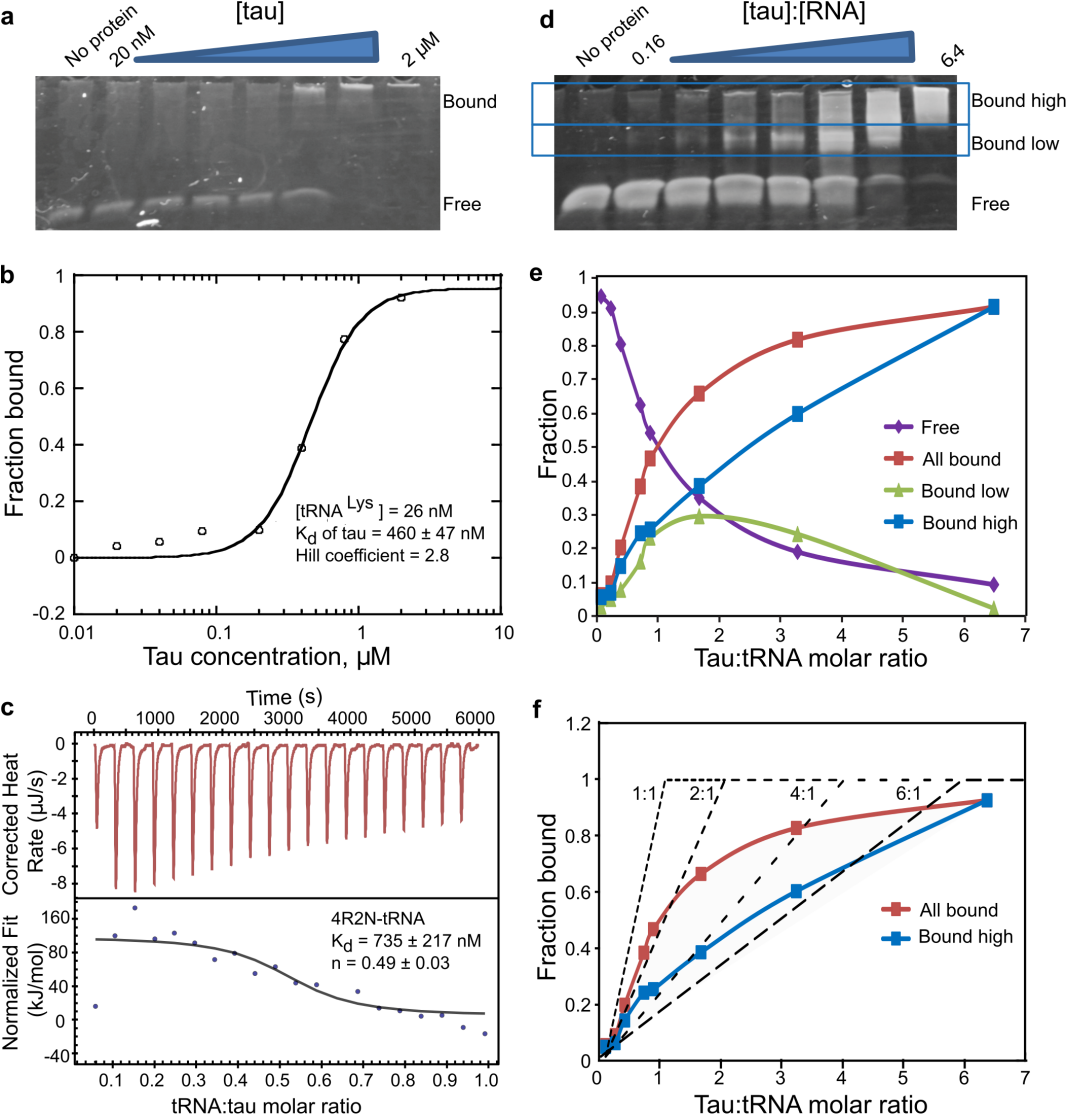
Tau tRNA binding by gel shift assay and isothermal titration calorimetry (ITC). (A) Direct titration experiment shows 4R2N tau induces a mobility shift in tRNA^Lys^. To determine the K_d_ value, direct titration experiment was done, which requires trace RNA concentrations to meet the assumption used in the equation that derives the K_d_ value. The assumption is that the total protein concentration is approximately the free protein concentration at equilibrium, and that protein binding to RNA is negligible. In the direct titration experiments 26 nM, tRNA was used and protein concentration spans from 20 nM to 2 μM. (B) The fraction of bound tRNA by 4R2N tau plotted as a function of the monomeric tau concentration and fit to the Hill equation, y = 1 / [1 + (K_d_ / x)^n^]. (C) Yeast tRNA was titrated into solutions of 4R2N tau in an ITC experiment. The top panel shows the raw incremental-titration. The area under each peak is integrated and plotted against the tRNA:tau molar ratio and fitted to an independent binding model (the bottom panel), as discussed in Materials and methods. (B-C) SEM is reported from *n* = 3. (D) The stoichiometric binding experiments were performed by varying the tau:RNA molar ratio at a constant 2.6 μM concentration of tRNA, which approximates the saturation concentration (more than 5 times the K_d_ of 460 nM). The representative data of 3 independent experiments are shown. (E) Fraction of bound tRNA from the different bands in (D) is plotted over the molar tau:tRNA ratio. (F) Fraction of bound tau plotted as a function of tau:tRNA molar ratios and compared to the theoretical saturation binding curves (dotted lines) with protein:RNA molar stoichiometries of 1:1 to 6:1. The theoretical curves serve the purpose showing that multiple tau molecules bind tRNA with increasing tau concentrations, while the model is not meant to fit the data, given that multiple populations with different tau:tRNA ratios will coexist. The numerical data used in (B), (C), (E), and (F) are included in S1 Data.

A gel shift assay using recombinant wild-type, full-length human tau (4R2N) induced a shift in unacetylated tRNA^Lys^ (Fig 3A), yielding a dissociation constant (K_d_) for 4R2N tau binding to tRNA of 460 ± 47 nM (Fig 3B). The derived Hill coefficient [36] was 2.8, implying cooperative binding of multiple tau proteins to tRNA. Isothermal titration calorimetry (ITC) experiments independently confirmed the affinity of tau binding to tRNA to yield K_d_ = 735 ± 217 nM and 372 ± 9 nM for 4R2N and K18 tau, respectively (Fig 3C and S5A Fig). The dissociation constant for 4R2N binding to a random 43 nucleotide RNA sequence still yielded a K_d_ = 832 ± 94 nM with a Hill coefficient of 2.6 according to a gel shift assay, suggesting that tau effectively and nonspecifically binds RNA in vitro, although there may be differences in binding affinity.

The gel shift assay showed multiple bands corresponding to different tau:tRNA stoichiometries corresponding to high molecular weight protein-RNA complexes (Fig 3D, similar gel shift with the tau fragment K18 shown in S5B Fig). The fraction of bound tRNA to 4R2N tau (from the low and high bands) was plotted as a function of tau:tRNA molar ratios (Fig 3E), and compared to theoretical binding saturation curves representing a range of stoichiometries from 1:1 to 6:1 (see Fig 3F). The theoretical curves serve the purpose of showing that multiple tau molecules bind tRNA with increasing tau concentrations. The model is not intended for data fitting; rather it shows that multiple populations with different tau:tRNA ratios coexist. However, the data suggest that at lower tau:tRNA molar ratios, tau and RNA predominantly form protein dimer-RNA (P_2_R) complexes, while at higher tau:tRNA molar ratios, tau and tRNA form larger protein multimer-RNA (P_2n_R) complexes with 2*n* reaching as high as 6. This implies that tRNA is capable of binding multiple tau proteins in a multistep process. Interestingly, when the tau:tRNA ratio was decreased by increasing the tRNA concentration relative to tau, the higher order P_2n_R complexes dissociated to P_2_R, maintaining a tau dimer bound to RNA in the presence of excess (7.5-fold) RNA (S5C Fig). Such higher stoichiometric signatures were not observed in the ITC measurement, which is less sensitive to binding events associated with small changes in heat. However, ITC titration experiments showed that both 4R2N and K18 tau interacted with tRNA with the stoichiometry of a protein dimer (S5A Fig, *n* = 0.49 ± 0.03 and Fig 3C, 0.52 ± 0.04). We conclude that the formation of P_2n_R complexes with *2n* exceeding 2 must be relying on weak interactions between multiple tau proteins and tRNA. A 2-stage model, in which a protein dimer-RNA (P_2_R) complex forms first followed by the formation of a protein multimer-RNA (P_2n_R) complex, is in fact consistent with the model for RNA binding to the protein AUF1 [37] and hnRNP A1 [38].

### Tau phase-separates in the presence of RNA

**Fig 4:**
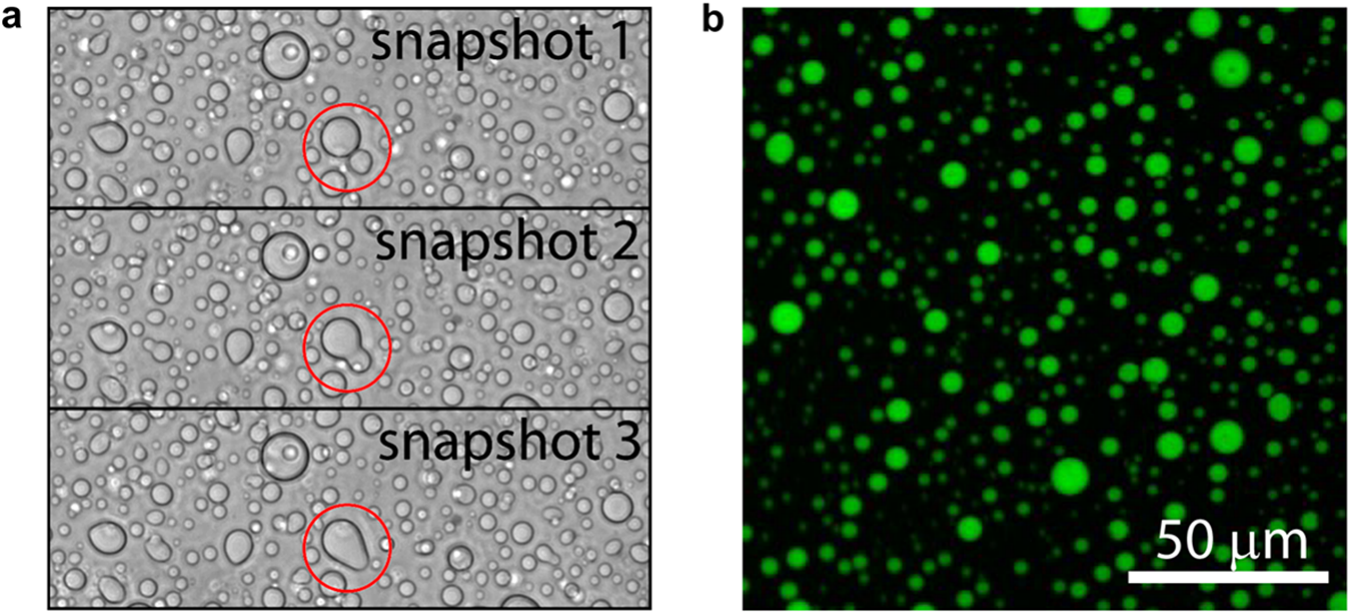
Tau and RNA forms droplet in vitro. (A) Bright field snapshots of droplets from 400 μM Δtau187 and 800 μg/ml poly(A) showing 2 droplets seamlessly fusing (highlighted with red circle). (B) Confocal microscopy image of Δtau187 labeled with Alexa-488 of the droplets from the same sample as in (A). 50 μM Alexa-488 labeled Δtau187 mixed with 350 μM MTSL labeled Δtau187 of droplets formed immediately after adding 800 μg/ml poly(A) RNA. Both (A) and (B) were sampled at room temperature and without added NaCl.

Mixing of 4R2N or Δtau187 (similar to K18, see S1 Fig) with tRNA (25 kDa), poly(A) RNA (66~660 kDa), or poly(U) RNA (800~1000 kDa) reliably produced a turbid solution under a wide range of tau:RNA mass ratios and salt concentrations. According to bright-field microscopy (Fig 4A), droplets formed and phase separated from the bulk aqueous phase with a clearly visible and highly spherical boundary. Tau droplets were capable of merging into a single droplet with the complete and nearly instantaneous loss of any boundary at the fusion interface, indicating that the droplets are fluidic with a relatively low interfacial tension (a series of snapshots capturing the fusion of 2 droplets are shown in Fig 4A). Confocal microscopy images of fluorescence-labeled tau verified that tau was predominantly contained within the droplet (Fig 4B). Depending on the specific condition, we were able to observe droplet formation with total Δtau187 protein concentration ranging between 2–400 μM, as will be discussed in greater detail next.

### Tau-RNA droplets form a complex coacervate phase

**Fig 5:**
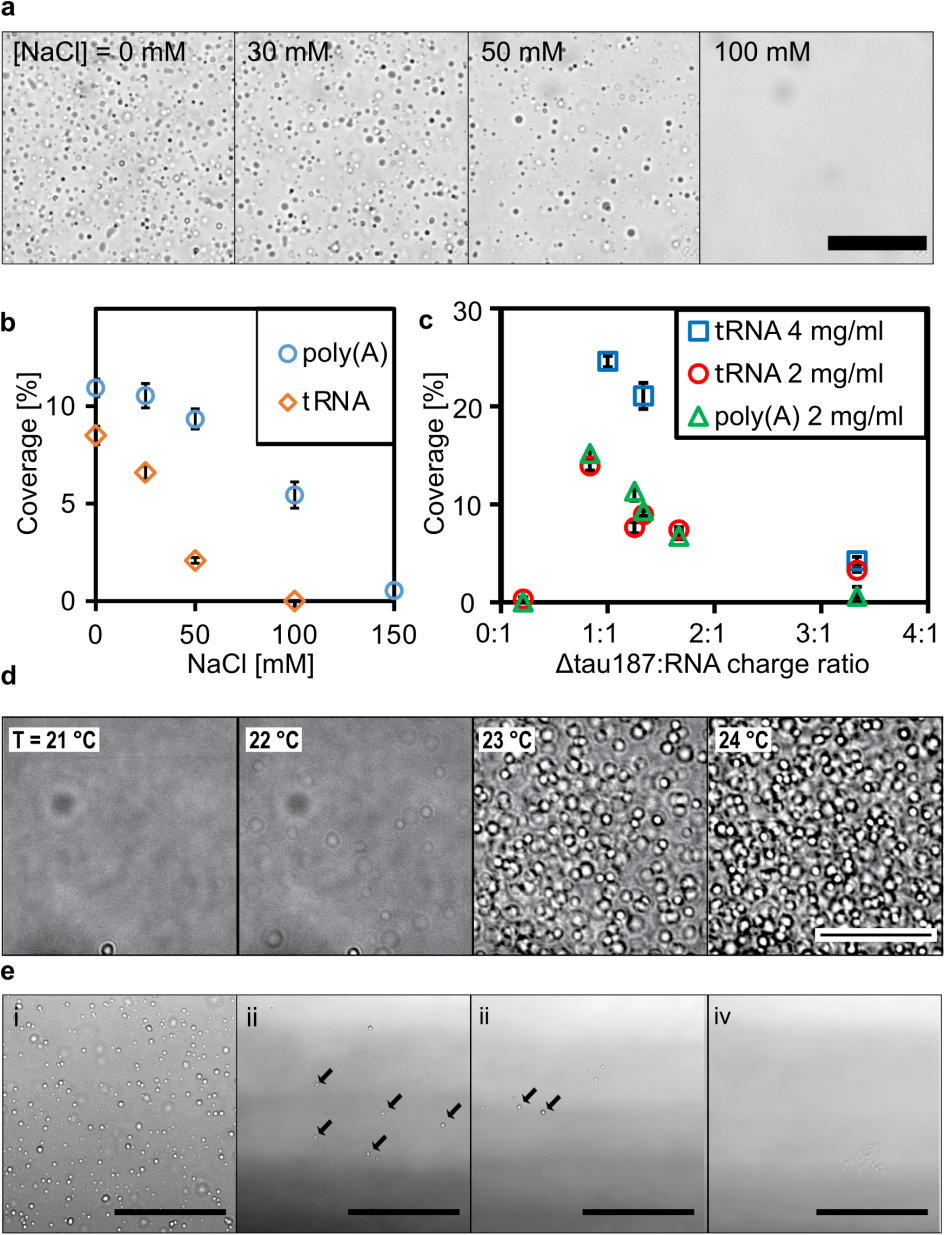
Tau-RNA droplets form a complex coacervate phase. (A) Representative bright-field images of tau-tRNA droplet samples at room temperature with varying [NaCl]. (B) Droplet coverage (in %) with poly(A) or tRNA in room temperature with varying [NaCl]. Δtau187:RNA in (A) and (B) were maintained at a mass ratio of 7:1 (corresponding to a charge ratio of 1.2:1) and a total mass concentration of 2 mg/ml. (C) Droplet coverage with poly(A) or tRNA with varying Δtau187: RNA charge ratios. The total mass concentrations are indicated in the legends. Samples made by mixing of 80 μM Δtau187 with 222 μg/ml poly(A)/tRNA or 161 μM Δtau187 with 444 μg/ml tRNA gave the highest droplet coverage (%), which correspond to charge ratio of 1.3–1.2 between tau and RNA. Error bars in (B) and (C) represent the standard deviation from *n* = 3. (D) Representative bright-field images of tau-RNA droplets as a function of incubation temperature. To record these images, the temperature was ramped from 19ºC to 25°C at 1°C per minute to acquire confocal images with bright field illumination. The samples for these images are generated from 100 μM tau mixed with poly(U) at approximately 1:1 charge ratio in the presence of 30 mM NaCl. (E) Representative bright-field images of tau-RNA droplet samples incubated at 37°C under otherwise different sample conditions. The concentration for tau, poly(U) RNA and NaCl are (i) 5 μM, 15 μg/ml, 0 mM; (ii) 5 μM, 15 μg/ml, 100 mM; (iii) 2.5 μM, 7.5 μg/ml, 100 mM; (iv) 1 μM, 3 μg/ml, 100 mM. Arrows highlight some of the droplets in images (ii) and (iii). Through Fig 5, scale bars are for 50 μm and all samples were prepared with Δtau187/322C in 20 mM ammonium acetate at pH 7.0. The numerical data used in (B) and (C) are included in S1 Data.

The amount of tau-RNA droplet was quantified as percent coverage from the microscopy images, as well as verified against turbidity measurements relying on light scattering at λ = 500 nm. Droplet formation follows spontaneously from the mixing of 2 oppositely charged biopolymers, tau and RNA, at a given range of pH, salt, and protein concentration, as well as temperature (Fig 5). This process is fully reversible and reproducible, characteristic of equilibrium states. We highlight the effect of salt concentration first. At a given total protein concentration and temperature, the systematic increase in salt concentration decreased the amount of tau-RNA droplet. Specifically, at room temperature, a total protein concentration of 80 μM and in the presence of tRNA, the droplets reproducibly disappeared when the salt concentration was increased to 100 mM or higher (see Fig 5A). This general trend was observed repeatedly under a range of experimental conditions, including the use of different RNA types (Fig 5B and S6A Fig, left panel). Interestingly, droplets formed in the presence of poly(A) and poly(U) tolerated higher than 100 mM salt concentration compared to tRNA. The amount of tau-RNA droplet was also found to be sensitively and reversibly tunable by temperature (Fig 5D). A sharp dependence of droplet formation on temperature can be seen in the series of images shown in Fig 5D, where droplets dissolve below 22°C and appear at and above 23°C. The observation of a clear increase in the amount of droplet with increasing temperature signifies an entropy-driven process. Consequently, when the temperature was increased to physiological conditions (37°C), droplet formation was observed at protein concentrations as low as 2.5–5 μM, in the presence of salt concentrations as high as 100 mM and glycerol added as a crowding reagent to mimic the intracellular environment (S6B Fig). This observation is illustrated in a series of images presented in Fig 5E—droplets are observed at NaCl concentration as high as 100 mM and tau concentrations as low as 2.5 μM when the temperature is elevated to 37°C, while maintaining the tau:RNA molar ratios. The quantity of droplets decreased with decreasing total tau concentration, and disappeared when tau concentration dropped from 2.5 to 1 μM (Fig 5E, panel iv). Given that the intracellular concentration of tau is approximately 2–4 μM in neurons [39, 40], conditions under which droplets were observed correspond to protein concentration, salt concentration, and temperature resembling physiological conditions.

Droplet formation was maximal, as measured by percent coverage from the microscopy images, with Δtau187:tRNA molar ratios of 8:1, Δtau187:poly(A) RNA molar ratios from 33:1 to 330:1 and Δtau187:poly(U) RNA ratios from 267:1 to 333:1 (estimated from the molecular weight of RNAs, see S6C Fig). By computing the charge (see S1 Text) and the mass (as measured by the molarity) of the RNA and protein components in the droplet samples, we found that these different molar ratios converge to approximately a 1:1 charge ratios regardless of the type of RNA used, including poly(A) and tRNA species (Fig 5C) and poly(U) (S6A Fig, right panel), and that at different total mass concentration of tau and RNA. Droplet formation was also observed with full length tau 4R2N and RNA at room temperature, but was less robust than with Δtau187, possibly due to the additional negative charges at the N-terminus of tau 4R2N that would diminish the electrostatic association between tau and RNA. However, at pH 6 where the net charge of tau 4R2N is similar to Δtau187, droplets reliably formed (S6D Fig), albeit with a significantly lower yield (S6E Fig)—note the percent coverage with tau 4R2N-derived droplets of order <3% compared to that with Δtau187-derived droplets of order 5%–30%.

The observations that droplet formation is directly, sensitively and reversibly tunable by salt concentration, tau:RNA charge ratios, as well as temperatures demonstrate that this process is modulated and toggled by both electrostatic interactions and changes in net entropy, and the balance between these contributions. Based on these findings, we conclude that tau-RNA droplet formation follows a complex coacervation mechanism as initiated through nonspecific and relatively weak complexation of oppositely charged and polyelectrolytes and driven by the further association of these polyelectrolyte complexes to form a macroscopic fluid phase.

### Tau in condensed droplets assumes solution state properties

**Fig 6:**
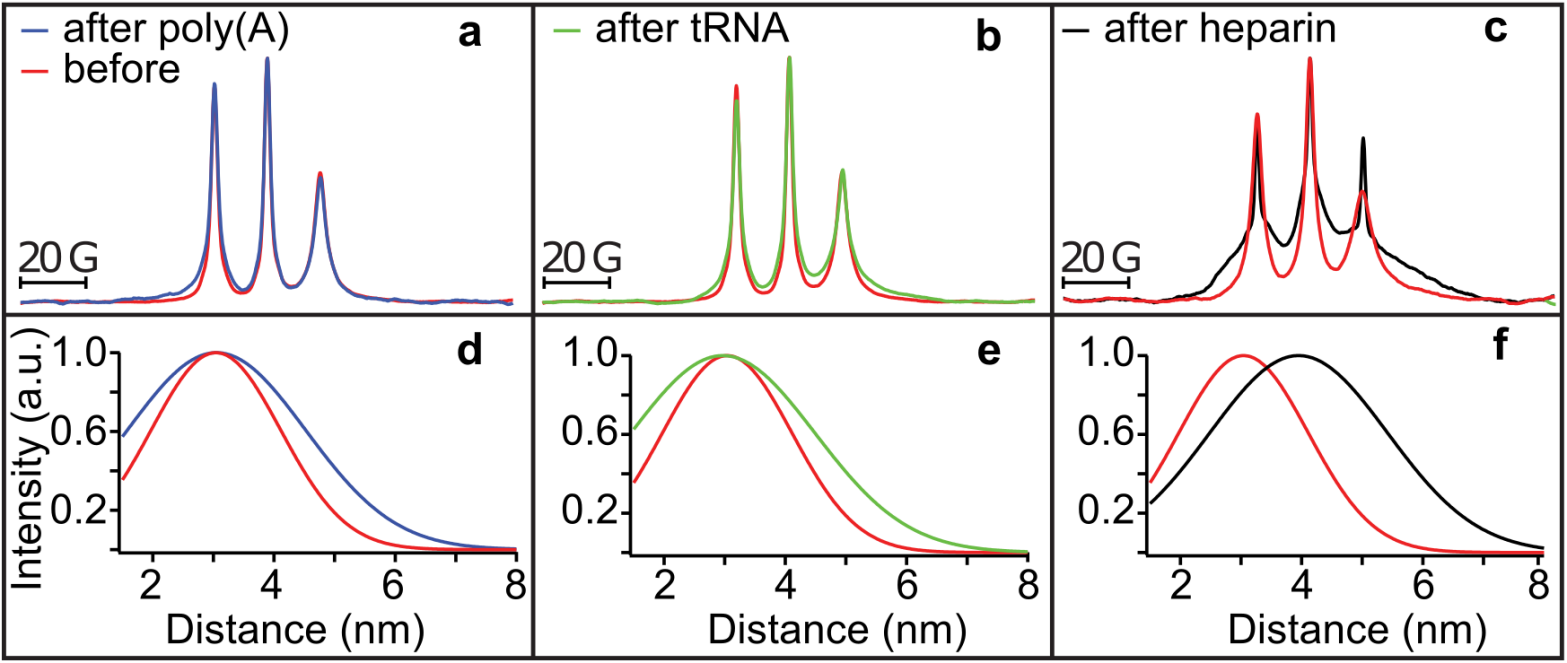
Tau in condensed droplets assumes solution state properties. (A-C) Continuous wave electron paramagnetic resonance (cw EPR) spectra obtained at room temperature of 500 μM Δtau187-SL in droplets formed with 1.5 mg/ml poly(A) RNA (blue in A) and 1.5 mg/ml tRNA (green in B) is unaltered from solution before adding RNA (red in A and B). Cw EPR line shape upon adding 125 μM heparin (black in C) show dramatic line broadening (compared to red in C). Double electron-electron resonance (DEER) of Δtau187-SL_2_ in droplets formed with 1.5 mg/ml poly(A) RNA (blue in D) and 1.5 mg/ml tRNA (green in E), as well as upon incubation with 137.5 μM heparin (black in F) compared to Δtau187-SL_2_ in solution (red in D-F). The numerical data are included in S1 Data.

Within a condensed complex coacervate fluid, held together by nonspecific and weak electrostatic interactions, we expect the polyelectrolyte constituents to maintain their hydration layer and remain dynamic [20, 41]. However, this assumption needs experimental verification. To spectroscopically track tau exclusively from within the condensed phase, we first verified by confocal fluorescence imaging that Δtau187 was predominantly localized within the droplet (see Fig 4B). This allowed us to characterize the droplet-internal protein properties by using spin labeled Δtau187. We carried out continuous wave electron paramagnetic resonance (cw EPR) spectral line shape analysis of singly spin labeled Δtau187 at a cysteine site 322, Δtau187/322C-SL, diluted with diamagnetically labeled Δtau187/322C-DL (see Materials and methods) to compare the protein side chain dynamics and degrees of freedom of the tethered spin label of Δtau187 in dilute solution state (Fig 6A–6C, red), in the droplet state associated with poly(A) RNA (Fig 6A, blue) or tRNA (Fig 6B, green), and upon addition of the tau aggregation inducer, heparin (Fig 6C, black). Remarkably, the EPR lineshape of Δtau187/322C-SL within the droplet phase was indistinguishable from that in the dilute solution state (e.g., see red versus blue trace in Fig 6A). In contrast, the EPR line shape dramatically broadened within minutes of adding heparin—a highly effective aggregation inducer of tau [1, 42] (e.g., see red versus black trace in Fig 6C). This finding experimentally demonstrates that the condensation of Δtau187 to high protein concentration, as found within droplets, alone is insufficient to induce dehydration and aggregation of tau, and that tau retains the protein dynamical properties as found in solution state, despite forming long-range associations with RNA in a highly concentrated fluid phase. From this, we infer that tau maintains its protein dynamics and hydration shell as in dilute solution state.

To determine whether tau assumes altered protein conformations in the complex coacervate phase compared to the dilute solution phase, we compared the intraprotein distances flanking the PHF6 and PHF6^*^ hexapeptide regions of tau under these conditions. These regions of the tau sequence pack into the β-sheet core once fibrils are formed of tau [43]. Recently, we identified that both the PHF6 and PHF6^*^ hexapeptide regions of tau undergo a dramatic opening from a compact to a fully extended local conformation, well before fibril formation is observed, and remain extended as they pack into β-sheet structures as part of insoluble fibrils [27]. These local distance measurements of the regions flanking the PHF6^(^*^)^ regions offer a powerful tool to compare the conformational state of tau in dilute solution, droplet and aggregation-induced states. We prepared a Δtau187 G272C/S285C-SL_2_ construct that was doubly MTSL-labeled at sites 272 and 285 (see Materials and methods) to access the distances across the PHF6^*^ hexapeptide region by double electron-electron resonance (DEER) spectroscopy. Surprisingly, the mean distance flanking the PHF6* region remained unchanged from dilute solution state to when tau was condensed into a concentrated complex coacervate phase in association with poly(A) RNA and tRNA (compare blue versus red trace in Fig 6D, and compare green versus red trace in Fig 6E). This contrasts with the effect of heparin on tau that markedly extended the mean distance between the labels (from approximately 3 to 4 nm) within minutes of heparin addition as recently reported [27], corresponding to the extended conformation that the PHF6^(*)^ segment adopts when neatly stacked in β-sheets (compare black versus red trace in Fig 6F).

Interestingly, we observed a low-level Thioflavin T (ThT) fluorescence under tau-poly(U) RNA droplet forming conditions that gradually increased over 15 hours (see Δtau187 + poly(U)). The ThT fluorescence, however, was nearly eliminated at increased salt concentration that corresponded to conditions under which tau-RNA associations are weakened and droplets are dispersed. However, even after 15 hours of incubation, the ThT fluorescence intensity from the tau-RNA droplet samples was significantly less (less than 5%) compared to what was observed in the presence of the aggregation-inducing heparin, under similar charge ratio and mass concentration and following a brief incubation time of less than 20 minutes (see S6F Fig, the histogram for Δtau187 + poly(U) versus heparin is not to scale, as indicated with a split y axis). Since ThT fluorescence is commonly used as an assay to detect β-sheet content, we suggest that droplet formation through association with RNA increases the aggregation propensity of tau in vitro, even when the tau-RNA complexes are held together by reversible and weak interactions between intact RNA and the hydrated tau constituents.

### Exogenous tRNA can induce sarkosyl insolubility of tau

**Fig 7:**
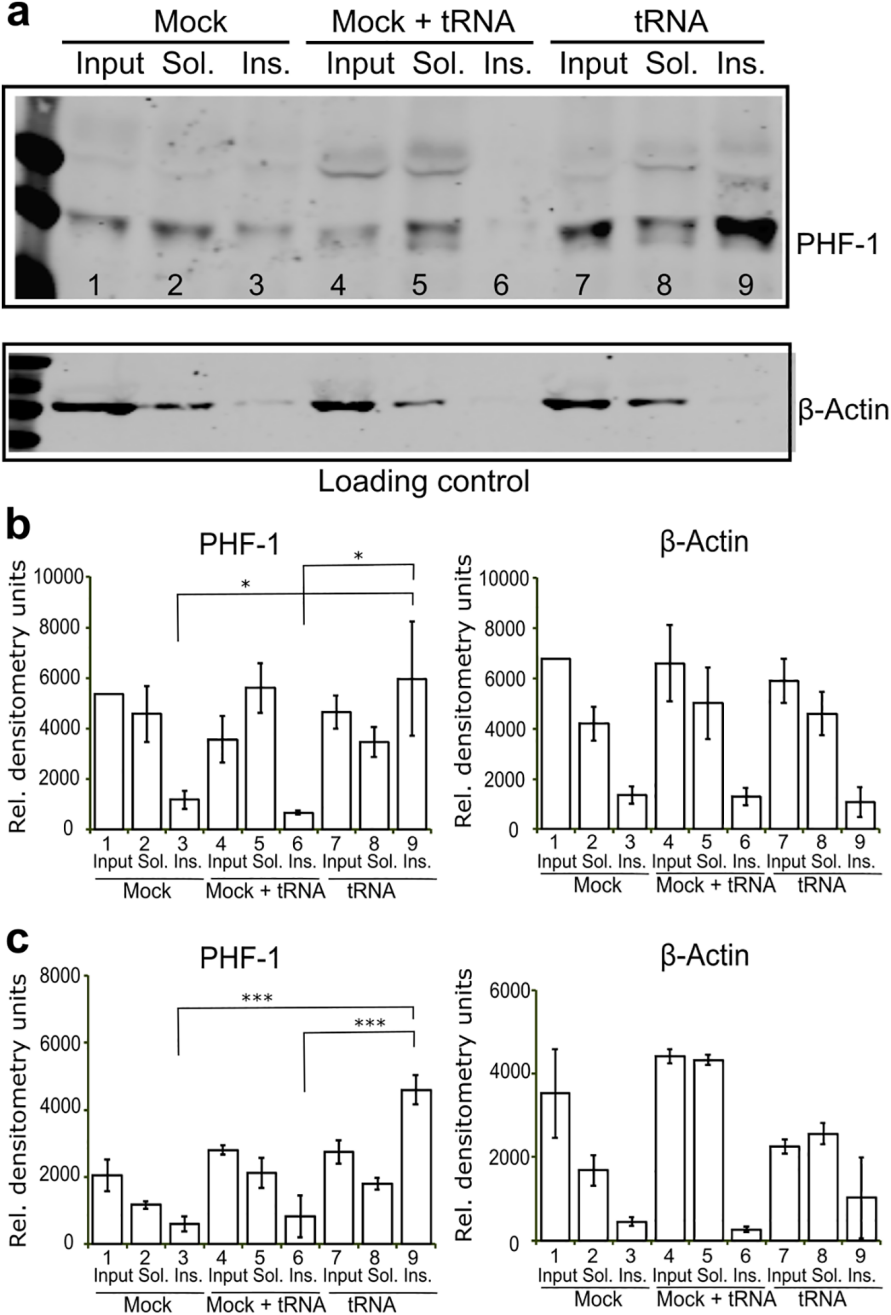
tRNA transfection accumulates sarkosyl insoluble tau in human-induced pluripotent stem cell (hiPSC) derived neurons. (A) Representative western blot of neurons harboring a P301L *tau* mutation transfected with tRNA, but not cells transfected in the absence of nucleic acids (Mock) present evidence for accumulation of sarkosyl insoluble tau (Ins.) as seen in the intense band in lane 9 labeled with PHF-1 antibody. Addition of tRNA to the lysis buffer is insufficient to increase the tau present in the insoluble fractions (compare Mock + tRNA, lane 6 to tRNA, lane 9). (B-C) Quantification of PHF-1 tau and β-actin level for both neurons harboring a P301L tau mutation (B) and neurons expressing wild type tau (C) are shown. Error bar represents standard error of the mean. * *p* < 0.05, *** *p* < 0.001, *n* > 3. The numerical data used in B and C are included in S1 Data.

To determine whether tau-RNA complexes have the potential for pathological interactions in vivo, hiPSC-derived neurons with a P301L mutation or wild type were transfected with 48 μg tRNA per 1.2 million cells. The uptake of tRNA was demonstrated with tRNA^Phe^ -fluorescein (S7 Fig). Cell lysates (input) were prepared in a high salt/high sucrose buffer, followed by fractionation in a 1% sarkosyl buffer. Transfection in the absence of nucleic acids (mock) or addition of tRNA to the mock lysate (mock + tRNA) were used as controls. Cells transfected with tRNA accumulated sarkosyl-insoluble tau as detected with the PHF-1 tau antibody; whereas cells without added tRNA, or when tRNA was added to the lysis buffer did not increase the tau population in the insoluble fractions (Fig 7A). The increase in sarkosyl-insoluble tau populations occurred in both P301L mutation and wild-type tau cells (Fig 7B and 7C) when infected with tRNA.

## Discussion

Many years ago, Wolde and Frenkel [44] proposed that condensed liquid droplets serve as a “metastable crucible” to lower free energy barriers for crystal nucleation, while the discovery of the involvement of nucleic acids in coacervation-driven LLPS dates back to 1949 by Bungenberg de Jong [21]. Here, we identify tau to undergo complex coacervation upon association with RNA. We find that tau can bind RNA in 2 stages, mediated by (1) strong binding at a tau:RNA molar ratio of 2:1 with nanomolar dissociation constants and (2) weak association between tau multimers and RNA to form higher order complexes. Although tau lacks a recognizable RNA-binding motif, it selectively bound tRNA as its dominant partner in vivo. Furthermore, tau was found to spontaneously phase separate upon nonspecific complexation with RNA into dense protein droplets, whose formation was reversibly and sensitively tunable by salt concentrations, tau:RNA ratios, as well as temperature. Crucially, the optimal tau:RNA molar ratios range from 8:1 to 330:1, which varied among different RNA species. Remarkably, all higher order complexes converge to a common tau:RNA charge ratio of approximately 1:1. This finding together with the sensitivity of droplet formation to salt concentration led us to conclude that tau-RNA droplet formation is driven by complex coacervation, in which oppositely charged polyelectrolytes associate into extended assemblies held together by electrostatic interactions. Significantly, EPR spectroscopy verified that tau remained locally dynamic, and maintained local conformations as found in solution state. This is consistent with an entropically driven complex coacervation process between tau and RNA, in which contribution from attractive tau-RNA interactions and changes in the biopolymer configurational entropy are small. We rationalize weak tau-RNA interaction to water-mediated electrostatic attraction between hydrated tau and RNA molecules, which is hence weakened. The total entropy of the complex coacervated system can be increased through the release of counter-ions and/or hydration water, provided that the entropy of the biopolymer constituents themselves remains relatively constant. That the release of hydration water may be one of the potential drivers of entropically driven complex coacervation is not contradictory to our statement that fully hydrated biopolymers constitute the complex coacervate phase. Even sharing the hydration shell between proteins in a crowded solution alone will increase the net entropy of the total system’s solvent, if the protein concentration within the coacervation is very high and the protein preserves its dynamics and hydration shell as in dilute solution state. Notably, there are several reports in the literature on the spontaneous complex coacervation of synthetic polyelectrolyte and protein (e.g., β-lactoglobulin) constituents to be entropy-driven [6, 18, 24, 45, 46], but no studies combine macroscopic thermodynamic measurements with studies of local protein dynamics and conformations. We show that the majority of tau complexed with RNA in the highly condensed droplet state maintains locally the dynamical conformations of free tau as populated in dilute solution state. In other words, tau can reversibly switch between the dilute solution and the dense droplet state involving minimal rearrangement of hydration water and protein conformations, as tuned by salt concentration, pH and temperature—i.e., physiologically viable “knobs.”

Still, a low-level ThT fluorescence was observed in the tau-RNA droplet samples after prolonged incubation, but with a significantly reduced amplitude compared to tau incubated with heparin. Consistent with this finding, tRNA increased sarkosyl insolubility of tau in vivo. We conclude that dynamic and free tau species are stored in a concentrated droplet state that can spontaneously and reversibly dissolves into solution at increased salt concentration and/or depressed temperature, while also being predisposed to converting to fibrils. This previously unrecognized droplet phase state contrasts with that of tau following incubation with the polyanion, heparin, in which a rapid conformational change occurs in solution state followed by an irreversible fibril formation. The distinguishing effects between RNA (charge density approximately 3.0/nm, estimated from the nucleic acid double helix [47], a polyphosphate) and animal-derived heparin (charge density approximately 4.0/nm, estimated from a disaccharide crystal structure [48], a polysulfate) on tau—coacervation versus fibrillization—may be an operational principle of tau-polyanion association.

Linking the in vitro observations of tau-RNA coacervation to in vivo structures that share many of the properties we describe here remains a challenge for the field. RNA granules [49–51], stress granules [52], germ line granules [53], synaptic vesicles [54], and chemical reaction centering at the origin of life [55] all lack taxonomy, and yet share features that resemble in vitro droplets. These structures are cohesively motile in the absence of any surrounding membrane, contain IDPs which often contain RNA-binding proteins and serve as an RNA transport vehicle in which translation is silenced [51, 56, 57]. TDP43, FUS, C9ORF72, hnRNPA2B1, and hnRNPA1 and TIA-1 [6–14] are all IDPs and RNA binding proteins, but establishing a connection between in vivo systems and the biophysics of droplets has been challenging. Brangwynne et al. have recently employed optogenetic control of Cry2 to trigger the association between IDPs tethered to Cry2 as a mechanism to induce phase separation involving the IDPs, presumably by increasing the local protein concentration. Our study not only adds tau to this prominent list of proteins that undergo LLPS upon association with RNAs, but also establishes a new phase state of tau in which soluble tau is reversibly stored in concentrated complex coacervate droplets, readily bioavailable for cellular functions or vulnerable to pathological aggregation depending on the environmental cues at hand, and offers guidance for the physiologically tunable factors that may promote LLPS in vivo—increased temperature, lower salt concentration, and exceeding a local threshold protein concentration.

## Materials and methods

### Cell culture

A total of 8 samples were used for PAR-iCLIP studies. Four samples were HEK (HEK 293T) cells that expressed either 4R2N (residues 1–441) wild-type *tau* (1 sample), 4R2N *tau* harboring the P301L mutation (1sample), and 4R1N *tau* harboring the P301L mutation fused to CFP (2 samples). Four samples were neurons derived from hiPSCs obtained by reprogramming dermal fibroblasts with virally transduced Yamanaka factors [58] that expressed wild-type *tau*, 2 harboring a P301L *tau* mutation, and 1 harboring the A152T variant [59, 60]. The hiPSC lines were karyotyped by Cell Line Genetics (Madison, WI). Both wild type and P301L were from normal males (46XY). A152T displayed a balanced 3-way translocation of chromosomes 1, 13, and 7, which most likely occurred during the reprogramming process (46XY, t(1;13;7) (q31.2;q21;q36.3)). Pluripotent cells were maintained under feeder-free conditions and cultured in BD Matrigel (BD Biosciences, San Jose, CA) coated 6-well plates and fed with mTSER daily (Stemcell Technologies, Vancouver, Canada). Neuroectoderm differentiation utilized dorsomorphin and SB431542 (Sigma-Aldrich, St. Louis, MO) for a week [61]. When neural-rosettes were clearly observable, the media was gradually replaced with neuronal induction media containing Knockout DMEM F12 with N2, and Glutamax 1X supplemented with laminin (Sigma-Aldrich, St. Louis, MO) and maintained for an additional 6 days with every other day feeding. Neuro-rosettes were microdissected, and grown as neurospheres in the neuronal induction media supplemented with B27 supplement. Neurons were differentiated from neurospheres by dissociating them into individual cells enzymatically with a 1/1 mixture of 0.25% trypsin/StemPro accutase (Thermo Fisher, Waltham, MA), and plated on Poly-L-Ornithin/Laminin coated 6 well plates at a density of 200,000 cells per well. Neuron differentiation and maturation were stimulated by the addition of 10 mM each of NT3, BDNF, and GDNF (Preprotech, Rocky Hill, NJ) to the neuronal growth media containing Neurobasal, N2 supplement, B27 supplement, and Glutamax. Neurons were fed twice per week by replacing half of the conditioned media with prewarmed supplemented fresh media, and underwent maturation for at least 5 weeks. At this developmental stage, hiPSC-derived neurons predominantly express the 3R tau isoform. Neuroblastoma (SH-SY5Y) cells were plated as monolayers in DMEM/F12 medium with 10% FBS at 1x 10^6^ per 10 cm dish, and the cells switched to neurobasal medium the next day and differentiated for 7 days using 10 μM retinoic acid.

### Antibodies

The tau antibodies used were Tau-5 for probing total tau in western blot (1 mg/ml, Abcam, Cambridge, UK); human tau-specific HJ 8.5 and mouse tau-specific HJ 9.2 (4.5 mg/ml and 2.8 mg/ml, both HJ antibodies are gifts from Dave Holtzman, Washington University) for CLIP; PHF-1 (gift from Peter Davis, Albert Einstein College of Medicine) for sarkosyl-insoluble tau. Other antibodies used are MAP2 antibody (0.3 mg/ml, Proteintech, Rosemont, IL); CDK5 rabbit antibody (0.2 mg/ml, Santa Cruz Biotechnology, Santa Cruz, CA); GFP rabbit antibody (5 mg/ml, Abcam, Cambridge, UK); β-Actin (Sigma-Aldrich, St. Louis, MO); mouse IgG_1_ Alexa 680, (2 mg/ml, Invitrogen, Carlsbad, CA); mouse IgG Alexa 680 (2 mg/ml, Invitrogen, Carlsbad, CA); rabbit IgG 800 (1 mg/ml, LI-COR, Lincoln, NE).

### PAR-iCLIP

PAR-iCLIP was applied to detect specific RNAs bound to tau in living cells [30, 32]. Cells were treated with 4-thiouridine (4SU) (Sigma-Aldrich, St. Louis, MO) for 1 hour at a final concentration of 500 μM at 37°C, rinsed with ice-cold 1x PBS and irradiated one time with 400 mJ/cm^2^ of 365 nm UV light on ice. 4-SU can enhance the cross-linking efficiency especially for proteins in the cytoplasm [29, 30, 32]. The cells were centrifuged and the pellet stored at −80°C. The major steps of PAR-iCLIP are listed in S8 Fig and detailed in the S1 Text. CLIP experiments require an antibody, which can effectively immunoprecipitate tau under stringent high salt wash conditions. HJ 8.5 [62] raised against human tau efficiently depleted tau. With a dissociation constant of 0.3 pM, HJ 8.5 pulled down tau under the high-stringency purification conditions of CLIP and remained bound to tau throughout the procedure. Control experiments with GFP or CDK5 antibody for the lysate expressing those proteins, or with HJ 8.5 antibody for lysates without expressing tau were always done in parallel to rule out false-positive binding caused by the beads. After immunoprecipitation, the cross-linked RNA was radiolabeled, the protein separated on a SDS-PAGE gel, and transferred to nitrocellulose membrane and blotted. The RNA-protein complexes from CLIP experiments (Fig 1A–1C) were cut from the blot, and the RNA extracted followed by reverse transcription for library preparation [30]. The ^32^P-labeled RNA band was run on a polyacrylamide Tris-Borate Urea (TBU) denaturing gel to demonstrate and confirm the size of the complex.

### Library preparation, deep sequencing and bioinformatics analysis of iCLIP

The iCLIP libraries contained an experimental and a random barcode, which allowed multiplexing and the removal of PCR duplicates. After the barcodes were introduced, sample and control from 1 set of experiments were mixed to remove batch-to-batch variation. Libraries were sequenced on an Ion Torrent Proton (Thermo Fisher, Waltham, MA). Fastx collapser from FASTX-Toolkit was used to collapse reads and filter replicates resulting from the PCR based on the random barcode. Reads were separated into samples by the barcodes at the 5ʹ ends of reads. The reverse transcription primers sequenced at both ends of the reads were trimmed with Cutadapt 1.9 (https://github.com/marcelm/cutadapt/) [63]. After trimming the barcodes, reads with 18 bps or more were kept and counted as total unique reads, and aligned to the human genome (hg19) by Bowtie2 2.2.6 (http://bowtie-bio.sourceforge.net/bowtie2/index.shtml) [64]. RseQC [65] was used to evaluate the quality of sequencing and mapping reads. Alignments with scores equal to or greater than 10 were kept for downstream analysis. Reads from these RNA pools were clustered by their alignments, and Pyicos tools [66] were used to identify the significant clusters. Clusters with at least 5 reads were retained and considered to contain target sites for RNA-tau crosslinking. Gene models for RefSeq mRNAs, tRNAs, and rRNAs were downloaded from the UCSC genome browser. miRNAs were downloaded from miRBase (release 20) and other categories of RNAs were download from Ensembl (release 73). Cross-link sites were identified as the termination site of the sequencing based on the iCLIP protocol [67]. Individual tRNA genes from the UCSC genome browser were predicted by using tRNAscan-SE v.1.23. The secondary structure for each tRNA was obtained from GtRNAdb (http://gtrnadb.ucsc.edu/).

### Deep sequencing and analyses of small RNA expression in HEK cells and hiPSC-derived neurons

Small RNAs were extracted from HEK cells and hiPSC-derived neurons using miRNA isolation kit (mirVana; Thermo Fischer, Waltham, MA). Library preparation was adapted from the Ion RNA-seq v2 (Thermo Fisher, Waltham, MA) protocol. cDNAs with size range from 30–100 bp were selected and sequenced. Reads were aligned to both human genomes and tRNA sequences. When mapping reads to tRNA sequences, the Bowtie read aligner [68] protocol was used, in which a maximum of 2 mismatches were allowed. Reads aligned to tRNAs were counted and analyzed with a custom Perl scripts.

### Recombinant tau and tau fragments

Full-length recombinant human 4R2N tau, N-terminal truncated, microtubule binding domain containing, K18 tau (residues 244–372), and Δtau187 (residues 255–441 with a His-tag at the N-terminus) were used for in vitro studies. Methods for expression and purification of all recombinant tau variants are detailed in S1 Text. Two variants of Δtau187 were prepared via site-direct mutagenesis: Δtau187/322C contains a C291S mutation, leaving only 1 cysteine at site 322, and Δtau187G272C/S285C contains C291S, C322S, G272C, and S285C mutations, leaving 2 cysteines at sites 272 and 285.

### RNA gel mobility shift assay

The gel shift assay was performed with recombinant full length 4R2N and K18 tau and chromatographically purified unacetylated yeast tRNA^Lys^ (tRNA Probes) in 100 mM sodium acetate buffer at pH 7.0. The molar concentration of tRNA^Lys^ was accurately remeasured with UV spectrophotometry after base-hydrolization to account for the hyperchromic effect from the secondary and tertiary structure of tRNA [36]. RNA43 was from Pierce RNA 3ʹ end biotinylation kit (Thermo Scientific, Waltham, MA) with the sequence of 5ʹ-CCUGGUUUUUAAGGAGUGUCGCCAGAGUGCCGCGAAUGAAAAA-3ʹ. The hydrolyzed tRNA and RNA43 samples were then quantified with UV spectrophotometry at 260 nm using an extinction coefficient of 0.025 (μg/ml)^-1^cm^-1^. For the gel shift assay, protein was incubated with tRNA at 37°C for 10 minutes in the presence of 0.5 mM EDTA, 0.5 mM MgCl_2,_ 2 U SUPERase• In RNase Inhibitor (Thermo Fisher, Waltham, MA), 0.01% IGEPAL CA-630 (Sigma-Aldrich), and then applied to a TBE 8% Polyacrylamide Gel (Thermo Fisher, Waltham, MA). After gel separation, tRNA was stained with SYBR Gold II (Thermo Fisher, Waltham, MA). For quantitative analysis, the fraction of free and bound tRNA was quantified in ImageJ2 (National Institute of Health, Bethesda, MD) [69].

### ITC experiments

Full-length tau or K18 tau were dialyzed overnight into a specified buffer for ITC (20 mM ammonium acetate, pH 7). tRNA (from Baker’s yeast; Sigma-Aldrich, St. Louis, MO) was resuspended in the ITC buffer and concentration determined by using a Nanodrop 1000 (Thermo Scientific, Waltham, MA). Experiments were run on a Nano ITC (TA Instruments, New Castle, DE), in which 300 μM tRNA was titrated (5 μl injections) into a 1 ml protein solution of 30 μM tau. Data were analyzed by using the NanoAnalyze v3.6 software (TA Instruments, New Castle, DE). After subtracting the heat generated by tRNA titration into an empty buffer, the experimental data were fitted well with an independent binding model, but not with a cooperative nor an independent 2-site model. Fitting to a cooperative or to an independent 2-site model gave a statistical error over 100% for the first Ka value. This could be due to the low enthalpy governing the binding event that is observed in the ITC data, and could possibly be resolved with increased concentrations of the reactants; here, tau and RNA. However, solubility limitations likely preclude these experiments from being realistically achievable. Of note, the second Ka value produced by the 2-site model is very similar to the values obtained from an independent model, suggesting that the predominant interaction is indeed the one we have described. Experiments were repeated in triplicates and standard error of the mean reported.

### Spin labeling of Δtau187

To achieve labeling with paramagnetic or diamagnetic probes, the protein was dissolved in 6 M guanidinium hydrochloride and labeled overnight at 4°C using a 20-fold molar excess of the spin label (1-oxyl-2,2,5,5-tetramethylpyrroline-3-methyl) methanethiosulfonate (MTSL; Toronto Research Chemicals, North York, Ontario, Canada) or the diamagnetic analog of MTSL (1-Acetoxy-2,2,5,5-tetramethyl-δ-3-pyrroline-3-methyl) methanethiosulfonate (Toronto Research Chemicals, North York, Ontario, Canada). Excess label was removed using a PD-10 desalting column (GE Healthcare, Little Chalfont, United Kingdom) equilibrated in a 20 mM ammonium acetate buffer at pH 7.0. The protein was concentrated using a 3 kDa Amicon centrifugal filter (Thermo Scientific, Waltham, MA). The final protein concentration was determined by UV-Vis absorption at 274 nm by using an extinction coefficient of 2.8 cm^-1^mM^-1^, calculated from extinction coefficient of Tyrosine [70]. The 2 variants Δtau187/322C and Δtau187G272C/S285C were spin labeled with paramagnetic MTSL probes at the 1 or 2 cysteine sites, and are referred to as Δtau187/322C-SL and Δtau187G272C/S285C-SL_2_, respectively. In order to achieve spin dilution, Δtau187/322C and Δtau187G272C/S285C were also labeled with the diamagnetic analogue of MTSL probes, and are referred to as Δtau187/322C/322C-DL and Δtau187G272C/S285C-DL_2_. Images shown in Fig 4A and 4B were taken by using 400 μM tau, 800 μg/ml poly(A) RNA and 30% glycerol in 20 mM ammonium acetate at pH 7.

### Preparation of tau-RNA complex coacervate

Droplets were formed in the 20 mM ammonium acetate buffer with NaCl concentration varying between 0 and 100 mM and glycerol concentration from 0% to 50% v/v. Unless explicitly indicated for measurements at various temperatures, all samples were prepared and characterized at room temperature. Solutions of Δtau187 or full length 4R2N tau was mixed with tRNA (Baker’s yeast; Sigma-Aldrich, St. Louis, MO), poly(A) RNA, or poly(U) RNA (Sigma-Aldrich, St. Louis, MO) at varying protein, RNA, NaCl, and glycerol concentrations in a 0.6 ml Eppendorf tube. RNAs were weighted out as powder and the mass concentration was calculated. The tau:RNA mass ratio, the total concentration of tau and RNA, as well as the NaCl salt concentration were optimized to maximize droplet formation, while choosing a total biopolymer density to avoid overlapping of droplets in the images to simplify the calculation of the droplet coverage (%). Microscopy images were acquired at 10 minutes after mixing—the droplets form spontaneously and are expected to be in equilibrium with the dilute supernatant. A concentration of 19% v/v for glycerol/water was determined to be an optimal concentration to ensure cryoprotection for DEER measurements carried out at approximately 80 K, while also ensuring maximal droplet formation at room temperature (S6B Fig).

### Bright-field microscopy of tau-RNA droplets

Immediately after mixing tau in a 0.6 ml Eppendorf tube with RNA under droplet forming conditions (established above) and ensuring thorough mixing, 1 μl of this mixture was applied to a microscope slide that is closed with a cover slide gapped by 2 layers of double-sided sticky tape to generate a liquid sample region with consistent thickness. The microscope slide was kept at room temperature for 10 minutes with the cover slide facing down, during which the particles within the liquid sample region settled down onto the surface of the cover slide. Images were taken with a 12-bit charge coupled device (CCD) camera across the entire sample liquid region near the surface of the cover slide using an inverted compound microscope (Olympus IX70; Olympus, Center Valley, PA). Before imaging, Köhler illumination was applied and the focus close to the surface of the cover slide optimized to enhance the contrast between the dark droplets and the bright background.

### Confocal microscopy of tau-RNA droplets

For confocal microscopy, Δtau187/322C was fluorescence-labeled (Δtau187/322C-FL) with Alexa Fluor 488 C_5_ Maleimide (Thermo Fisher) at the same 322 site as Δtau187/322C-SL. 50 μM of Δtau187/322C-FL was mixed with 350 μM Δtau187/322C-SL at a 1:7 molar ratio in order to prevent saturation. 800 μg/ml of poly(A) RNA was further added to this tau solution, resulting in droplet formation in the 20 mM ammonium acetate buffer and in the presence of 20 mM NaCl. 10 μl of the mixture was pipetted and put on a microscope slide with a cover slide gapped by double-sided sticky tape. Confocal images were acquired using a spectral confocal microscope (Olympus Fluoview 1000; Olympus, Center Valley, PA).

### Droplet quantification from image analysis

To quantify the amount of droplet formed under the given experimental condition of interest, images were taken by a 12-bit CCD camera of an inverted compound microscope (Olympus IX70; Olympus, Center Valley, PA), and recorded in TIF format. With illumination and focus optimized, droplets settling on the cover slide have lower intensity than their surrounding on the images. An image of the 20 mM ammonium acetate buffer was taken to calculate the average intensity to set as threshold in order to classify different parts of the image into droplets and buffer. For each image, the area of the droplets was divided by the total area of the image, generating a percent droplet coverage value on the cover slide. Droplets with eccentricity above 0.9 or equivalent diameter below 1μm were filtered out in order to reduce false reading. The MATLAB code is made available as supplementary files on the internet (https://github.com/yanxianUCSB/DropletAnalysis).

### cw EPR

Cw EPR relies on the anisotropy of nitroxide radical’s Larmor frequency and hyperfine coupling that makes its lineshape highly sensitive to the local dynamics, orientation and confinement of a nitroxide-based spin label tethered to the protein. Cw EPR measurements were carried out with Δtau187/322C-SL by using a X-band spectrometer operating at 9.8 GHz (EMX; Bruker Biospin, Billerica, MA) and a dielectric cavity (ER 4123D; Bruker Biospin, Billerica, MA). Samples were prepared by either mixing 200 μM Δtau187/322C-SL with 300 μM unlabeled Δtau187/322C (to generate 40% spin labeled sample) or by using 500 μM Δtau187/322C-SL (100% labeled). Viscogen was added to the sample to achieve either 19% v/v glycerol (for the droplet samples) or 30% v/v sucrose (for the aggregated samples) matching the DEER conditions. tau samples under droplet forming condition were prepared by adding 1.5 mg/ml RNA, and tau samples under aggregation-inducing conditions prepared by adding 125 μM heparin (11 kDa average MW, Sigma-Aldrich, St. Louis, MO). A sample of 3.5 μl volume was loaded into a quartz capillary (CV6084; VitroCom, Mountain Lakes, NJ) and sealed at 1 end with critoseal and the other with beeswax, and then placed in the dielectric cavity for measurements. Cw EPR spectra were acquired by using 2 mW of microwave power, 0.3 gauss modulation amplitude, 150 gauss sweep width, and 25 scans for signal averaging.

### DEER

DEER measurements were performed on a Q-band pulsed EPR spectrometer operating at 32 GHz (E580; Bruker Biospin, Billerica, MA) equipped with a QT2 resonator (measurements done courtesy of Bruker Biospin). Samples were prepared by mixing 50 μM Δtau187G272C/S285C-SL_2_ with 500 μM analog-labeled Δtau187G272C/S285C-DL_2_ at a 1:10 molar ratio to achieve spin-dilution and avoid artifacts from unwanted inter-protein spin distances. For DEER, tau samples under droplet forming condition were prepared by adding 1.65 mg/ml RNA and ensuring 19% v/v glycerol concentration, and tau samples under aggregation-inducing conditions prepared by adding 137.5 μM heparin and ensuring 30% v/v sucrose concentration. 40 μL samples containing 550 μM concentration of tau were loaded into a quartz capillary (2.4 mm od x 2 mm id) and flash frozen in liquid nitrogen after 20 minutes of incubation at room temperature and the specific conditions listed for the data. DEER measurements were conducted using the dead-time–free 4-pulse DEER sequence at 80 K, by using 22 ns (π/2) and 44 ns (π) observe pulses and a 30 ns (π) pump pulse. The raw DEER data were processed using Gaussian fitting via DeerAnalysis2013 (ETH Zürich, Zürich, Switzerland) [71].

### Quantification of fibril using ThT assay

160 μM Δtau187 was mixed with 480 μg/ml poly(U) RNA or 40 μM heparin and incubated at room temperature in the presence of 4 μM ThT for over 15 hours (S6F Fig). The fluorescence intensity at 485 nm was measured by using a plate reader (Tecan Infinite 200 Pro; Tecan, Männedorf, Switzerland).

### tRNA transfection

Transfection of RNA in neuron was first validated using tRNA^Phe^ -fluorescein transfected in primary mouse neuron at 14 days in vitro by using lipofectamine 2000 transfection reagent (Thermo Fisher, Waltham, MA) following the manufacturer's protocol (S7 Fig) Cell nuclei was stained with DAPI before fluorescence microscopic imaging. For sarkosyl experiments hiPSC neuronal cultures containing wild type or mutant tau were transfected with 48 μg of bovine liver tRNA (Sigma-Aldrich, St. Louis, MO) per 6 well plate. Control cells were transfected in equal conditions in the absence of nucleic acid (Mock transfection). Mock transfected cells were lysed as described below, with or without 48 μg tRNA added to the lysis buffer.

### Sarkosyl insolube tau isolation and western blotting

Separation of sarkosyl insoluble tau was done as described in the literature [72, 73]. Briefly, adherent neuronal cell cultures were lysed with an ice-cold high salt/high sucrose Tris HCl buffer (0.8 M NaCl, 10% Sucrose, 10 mM Tris HCl pH 7.4) containing a 1X Protease Inhibitor Cocktail, and a 1X Phosphatase Inhibitor Cocktail. Lysis proceeded at 4°C for 30 minutes before detachment with a cell scraper, followed by mechanical dissociation using a micropipette. Immediately afterwards, the lysates were centrifuged at 4°C 3,000 x g for 15 minutes in a microcentrifuge. The clear supernatants were collected and sampled (Input). Sodium lauroyl sarcosinate (Sigma-Aldrich, St. Louis, MO) was then added to the supernatants to a final concentration of 1%, followed by a brief vortexing. Samples were incubated at 4°C with continuous rocking. Samples were then centrifuged at 4°C for 2 hours at 170,000 x g in a Beckman Coulter 70.1 Ti rotor. Sarkosyl soluble supernatants (Sol.) were collected. Sarkosyl insoluble (Ins.) pellets were resuspended in 2X sample loading buffer (250 mM Tris HCl pH 6.8, 10% Glycerol, 10% SDS, 0.5% bromophenol blue, 20 mM DTT) and heated to 95°C for 10 minutes. The previously collected input and the sarkosyl soluble fractions were diluted with the same sample loading buffer, and heated under similar conditions. Proteins were separated on a 10% SDS-PAGE, transferred to Nitrocellulose membranes, and blotted with either PHF-1 or β–actin antibody. Western blot signal was detected with a LI-COR Odyssey imaging system (LI-COR Biosciences, Lincoln, NE) and quantified in ImageJ2 (National Institute of Health, Bethesda, MD) [69].

## Supporting information

S1 Fig Overview of tau constructs used in this study. Full length human tau (the longest isoform, 4R2N) comprise the N-terminal projection domain (residues 1–243), the 4-repeat microtubule binding domain (residues 244–372) and the C-terminal region (residues 373–441). The inserts near the N-terminus−N1, N2−and the second repeat−R2−can be alternatively spliced, giving rise to six isoforms. Two hexapeptide motifs−PHF6* and PHF6−at the beginning of R2 and R3 repeats are known to promote paired helical filament (PHF) aggregation. Cells expressing 4R2N wild type, or mutant (A152T or P301L), or 4R1N P301L fused to CFP were used in PAR-iCLIP study, with the mutation sites marked here with asterisks. Full length 4R2N tau, K18 tau (residues 244–372) and Δtau187 (residues 255–441 with a His-tag at the N-terminus) were used for *in vitro* RNA binding and droplet formation studies. Two variants of Δtau187 were used for EPR line shape analysis and DEER study: Δtau187/322C contains a C291S mutation, leaving only one cysteine at site 322, and Dtau187G272C/S285C containing C291S, C322S, G272C and S285C mutations, leaving two cysteines at site 272 and 285 for double spin labeling.
(TIFF)

S2 Fig Tau PAR-iCLIP. Phosphor images in the blue frame (a-d) show ^32^P-labelled RNA crosslinked to tau protein in HEK cells expressing tau (a-c) and in neuroblastoma with endogenous tau (d). The experimental conditions are indicated at the top of each lane: CLIP experiments with expressed P301L tau and immunoprecipitated (IP’ed) with human specific tau antibody (HJ 8.5 gift from D. Holtzman) (panel a, lane 2), CLIP experiments with expressed GFP and GFP IP (panel a, lane 1). CLIP experiments with expressed wild type (WT) tau and IP’ed with HJ 8.5 (panel b, lanes 2 and 3), control without UV crosslinking and IP’ed with HJ 8.5 (panel b, lane 1), control in cells that do not express tau and IP’ed with HJ 8.5 (panel b, lane 4). RNAse as indicated (panel b). CLIP experiments with expressed P301L tau fused to CFP and IP’ed with HJ 8.5 (panel c, lanes 1 and 2). Controls without UV and without expressed tau (panel c, lanes 3 and 4). RNAse as indicated did not change the migration of the tau RNA complex (panel c). CLIP experiments with antibody HJ9.2 (gift from D. Holtzman) against mouse tau (mTau), HJ8.5 antibody against human tau, MAP2 antibody and CDK5 antibody (panel d, lanes 1–4, respectively). To the left of each phosphor image are the western blots that utilize the same antibodies used for the CLIP IP: GFP and tau (panel a), tau (panel b and c), tau MAP2 and CDK5 (panel d). Below each phosphor image shown are the loading control western blots, where the lysates input for immunoprecipitation are probed by antibody Tau-5 and anti-actin. The RNA-protein complexes marked within the rectangles of panel a were cut from the blot for library preparation and sequencing. Note that two regions from the GFP control were cut and one corresponding to the MW of tau protein and other corresponding to the MW of GFP. (e) PAR-iCLIP-mapped reads from four samples of HEK cells harboring WT, P301L and two P301L-CFP tau. (f) PAR-iCLIP-mapped reads from four samples of hiPSC-derived neurons harboring WT, two P301L and one A152T tau. Data from the higher and lower MW band of the GFP or CDK5 controls are labeled as GFP-H, GFP-L, CDK5-H and CDK5-L, respectively. The non-cross sample is from the control without UV crosslinking. The numerical data used in e-f are included in S1 Data.
(TIFF)

S3 Fig Distribution of tRNA in PAR-iCLIP samples vs total endogenous tRNA. (a-b) tRNA abundance in CLIP samples vs total small RNA controls in HEK cells (a) and hiPSC-derived neurons (b) indicate that tRNA distributions differ between the total tRNA pool and the CLIP tRNA pool. Specifically, the population above the diagonal is much greater, indicating that the tRNA abundance from PAR-iCLIP samples from tau-bound RNA is significantly greater compared to the total small RNA present in HEK or hiPSC neuron cells. (c) Top 10 ranked tRNAs bound by tau in HEK cell and hiPSC-derived neurons. The numerical data used in a-b are included in S1 Data.
(TIFF)

S4 Fig Accumulative reads from all tRNAs recovered in tau PAR-iCLIP. Accumulative CLIP cDNA reads from all tRNAs recovered in tau PAR-iCLIP of HEK cell and hiPSC-derived neurons (top two graphs) in comparison to the total small RNA population (small RNA-Seq) in HEK cells and hiPSC-derived neurons (bottom two graphs). The anticodon is designated as position 1–3 for alignment purpose, and the tRNA structure is shown below the x-axis in one dimension. Note the distinct pattern of crosslinking sites around the anticodon region in the PAR-iCLIP reads from both the Hek cells and hiPSC neurons in contrast to the expected 5’ bias of the tRNAs from the small RNA-Seq population. The numerical data used are included in S1 Data.
(TIFF)

S5 Fig Additional data showing tau-tRNA binding by gel shift assay and ITC. (a) Yeast tRNA was titrated into solutions of K18 tau in an ITC experiment. The top panels show the raw incremental-titration data. The area under each peak is integrated and plotted against the tRNA:tau molar ratio and fitted to an independent binding model (the bottom panel). The numerical data are included in S1 Data. (b) Tau-RNA binding experiments with varying tau:RNA molar ratio, while keeping tRNA concentration at 2.6 μM and changing the K18 concentration from 2 to 12 μM. Lane 1 shows 4 μM tau 4R2N as reference, and lane 2–5 2 μM, 4 μM, 6 μM, 12 μM of K18, respectively. The lower band of the 4R2N tau-tRNA or K18-tRNA complex are marked with blue and red arrows, respectively. The mobility of the lower band of the K18-tRNA complex is faster than that of the 4R2N tau-tRNA complex. (c) Tau-RNA binding experiments with varying tau:RNA molar ratio, while keeping tau concentration constant at 4 μM and incrementally increasing the tRNA concentration from 0.07 to 30 μM. Lane 1 shows the no-tau control with tRNA concentration at 0.07 μM, lane 2–10 shows 4 μM K18 with 0.07, 0.13, 0.26, 0.66, 1.3, 2.6, 6.6, 20, 30 μM tRNA, respectively; lane 11, and 4 μM tau 4R2N with 1.3 μM tRNA as reference. The highly concentrated RNA in lanes 8–10 hindered the penetration of the SYBR Gold II staining reagent. The lower bands of the K18-tRNA complexes are marked with a blue rectangle and of the 4R2N tau-tRNA complexes with a blue arrow.
(TIFF)

S6 Fig Additional data about tau and RNA forms droplet *in vitro*. (a) Mixing Δtau187 with poly(U) RNA lead to droplet formation *in vitro*. Left: droplet coverage with varying NaCl concentration in samples made of 160 mM Δtau187 and 480 mg/ml poly(U). Right: droplet coverage by varying Δtau187:poly(U) charge ratios with no NaCl added. A total mass concentration of Δtau187 and poly(U) was kept at ~2 mg/ml, with 74 mM Δtau187 and 240 mg/ml poly(U) showing the highest droplet coverage (%). (b) Droplet coverage (%) of Δtau187 and poly(U) RNA with varying glycerol concentration. Experimental condition was maintained with 80 mM Δtau187 and 120 mg/ml poly(U), and with no added NaCl. (c) Calculations of tau-RNA molar or charge ratio, based on their chemical properties (top table), for various experimental conditions where droplet formation is observed (bottom table). The charge of the RNA and protein species at a given pH were estimated (following calculations discussed below in the S1 Text). (d-e) Mixing full length 4R2N tau with poly(A) RNA lead to droplet formation *in vitro*. (d) Representative bright-field images of 4R2N tau-RNA droplet at two NaCl concentrations. (e) Left: droplet coverage (%) with varying NaCl concentration, and at a charge ratio of tau 4R2N and RNA at 0.7:1. Right: droplet coverage with varying 4R2N tau:RNA charge ratios, with no NaCl added. A total polymer mass concentration ~3.6 mg/ml was maintained in the experiments in d-e, with 80 mM 4R2N and 349 mg/ml poly(A) showing the highest droplet coverage. All data and images in a-e were acquired 10 minutes after mixing of tau, RNA and 19% glycerol in a 20 mM ammonium acetate buffer at pH 7. (f) Extent of poly(U) induced fibrilization of Δtau187 measured by Thioflavin T assay compared to that of heparin of Δtau187. Pyrimidines can react with ThT and give strong background. For that reason, we used poly(U) to carry out this assay. Error bars show standard deviation from n = 3 in a-b, e-f and the numerical data used are included in S1 Data.
(TIFF)

S7 Fig The fluorescence microscopy image of neurons. Primary mouse neurons at 14 days *in vitro* were transfected with 8 μg of tRNA^Phe ^-fluorescein (green) per million cells using lipofectamine 2000. The images with tRNA^Phe ^–fluorescein staining are shown together with DAPI (blue) staining of the nuclei. The tRNA^Phe^-fluorescein and DAPI-stained images show the colocalization of tRNA in the cell. Scale bar is 20 μm.
(TIFF)

S8 Fig Key steps of PAR-iCLIP. Pipeline of PAR-iCLIP in identifying RNA-tau interaction in intact cells. The protocol of PAR-iCLIP can be found in the Materials and Methods section of the manuscript, as well as in S1 Text.
(TIFF)

S1 Text Supplementary text and figures. (DOCX)

S1 Data Excel spreadsheets of numerical values used to create the corresponding figures. (XLSX)

## Abbreviations

CDK5: cyclin-dependent kinase 5
CFP: cyan fluorescent protein
CLIP: Cross-Linking Immunoprecipitation
cw: continuous wave
DEER: double electron-electron resonance
EPR: electron paramagnetic resonance
GFP: green fluorescent protein
HEK: human embryonic kidney
hiPSC: human-induced pluripotent stem cell
IDPs: intrinsically disordered proteins
ITC: isothermal titration calorimetry
LLPS: liquid-liquid phase separation
lincRNAs: long intergenic noncoding RNAs
miRNA: microRNA
PAR-iCLIP: PhotoActivatable Ribonucleoside-enhanced Individual-nucleotide resolution UV Cross-Linking ImmunoPrecipitation and high-throughput sequencing
snRNA: small nuclear RNA
ThT: Thioflavin T
