# Supplementary figures and images for: RNA stores tau reversibly in complex coacervates
Source: PLoS Biol. 2017 Jul 6;15(7):e2002183. doi: 10.1371/journal.pbio.2002183 (PMC5500003; doi:10.1371/journal.pbio.2002183)

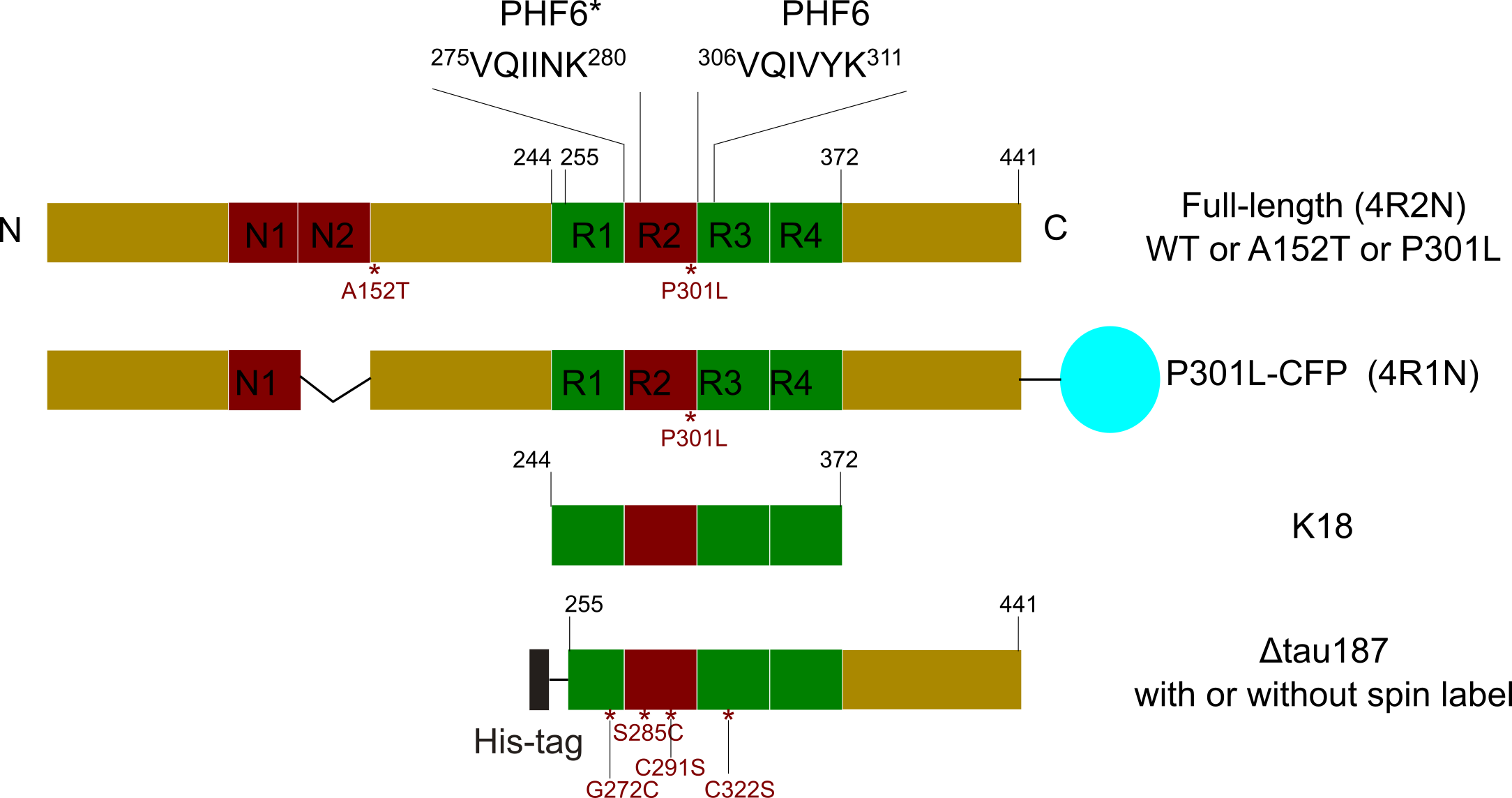

Supplement: S1 Fig — Full length human tau (the longest isoform, 4R2N) comprise the N-terminal projection domain (residues 1–243), the 4-repeat microtubule binding domain (residues 244–372) and the C-terminal region (residues 373–441). The inserts near the N-terminus−N1, N2−and the second repeat−R2−can be alternatively spliced, giving rise to six isoforms. Two hexapeptide motifs−PHF6* and PHF6−at the beginning of R2 and R3 repeats are known to promote paired helical filament (PHF) aggregation. Cells expressing 4R2N wild type, or mutant (A152T or P301L), or 4R1N P301L fused to CFP were used in PAR-iCLIP study, with the mutation sites marked here with asterisks. Full length 4R2N tau, K18 tau (residues 244–372) and Δtau187 (residues 255–441 with a His-tag at the N-terminus) were used for in vitro RNA binding and droplet formation studies. Two variants of Δtau187 were used for EPR line shape analysis and DEER study: Δtau187/322C contains a C291S mutation, leaving only one cysteine at site 322, and Dtau187G272C/S285C containing C291S, C322S, G272C and S285C mutations, leaving two cysteines at site 272 and 285 for double spin labeling. (TIFF) [file pbio.2002183.s001.tiff]

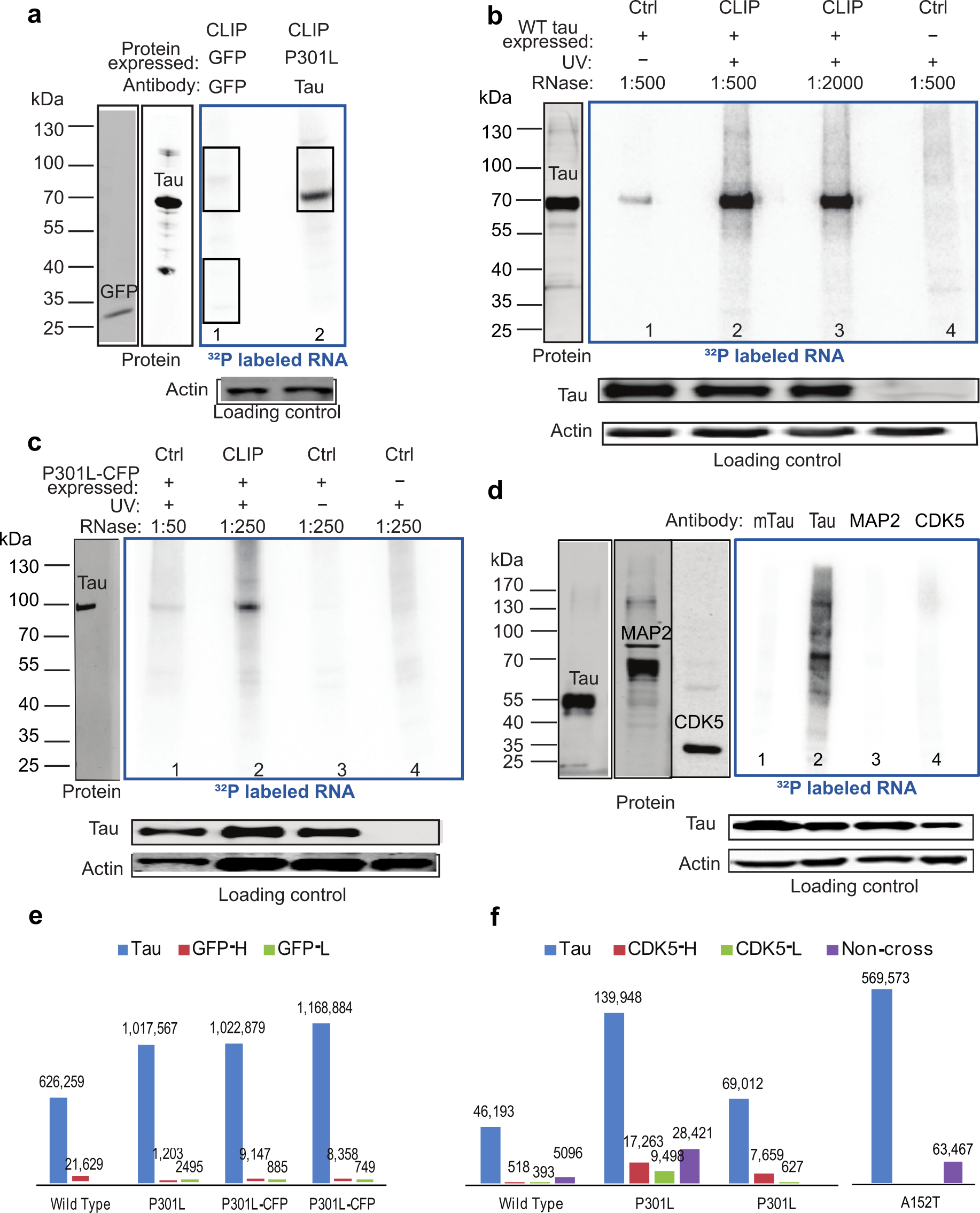

Supplement: S2 Fig — Phosphor images in the blue frame (a-d) show 32P-labelled RNA crosslinked to tau protein in HEK cells expressing tau (a-c) and in neuroblastoma with endogenous tau (d). The experimental conditions are indicated at the top of each lane: CLIP experiments with expressed P301L tau and immunoprecipitated (IP’ed) with human specific tau antibody (HJ 8.5 gift from D. Holtzman) (panel a, lane 2), CLIP experiments with expressed GFP and GFP IP (panel a, lane 1). CLIP experiments with expressed wild type (WT) tau and IP’ed with HJ 8.5 (panel b, lanes 2 and 3), control without UV crosslinking and IP’ed with HJ 8.5 (panel b, lane 1), control in cells that do not express tau and IP’ed with HJ 8.5 (panel b, lane 4). RNAse as indicated (panel b). CLIP experiments with expressed P301L tau fused to CFP and IP’ed with HJ 8.5 (panel c, lanes 1 and 2). Controls without UV and without expressed tau (panel c, lanes 3 and 4). RNAse as indicated did not change the migration of the tau RNA complex (panel c). CLIP experiments with antibody HJ9.2 (gift from D. Holtzman) against mouse tau (mTau), HJ8.5 antibody against human tau, MAP2 antibody and CDK5 antibody (panel d, lanes 1–4, respectively). To the left of each phosphor image are the western blots that utilize the same antibodies used for the CLIP IP: GFP and tau (panel a), tau (panel b and c), tau MAP2 and CDK5 (panel d). Below each phosphor image shown are the loading control western blots, where the lysates input for immunoprecipitation are probed by antibody Tau-5 and anti-actin. The RNA-protein complexes marked within the rectangles of panel a were cut from the blot for library preparation and sequencing. Note that two regions from the GFP control were cut and one corresponding to the MW of tau protein and other corresponding to the MW of GFP. (e) PAR-iCLIP-mapped reads from four samples of HEK cells harboring WT, P301L and two P301L-CFP tau. (f) PAR-iCLIP-mapped reads from four samples of hiPSC-derived neurons harboring WT, two P301 [file pbio.2002183.s002.tiff]

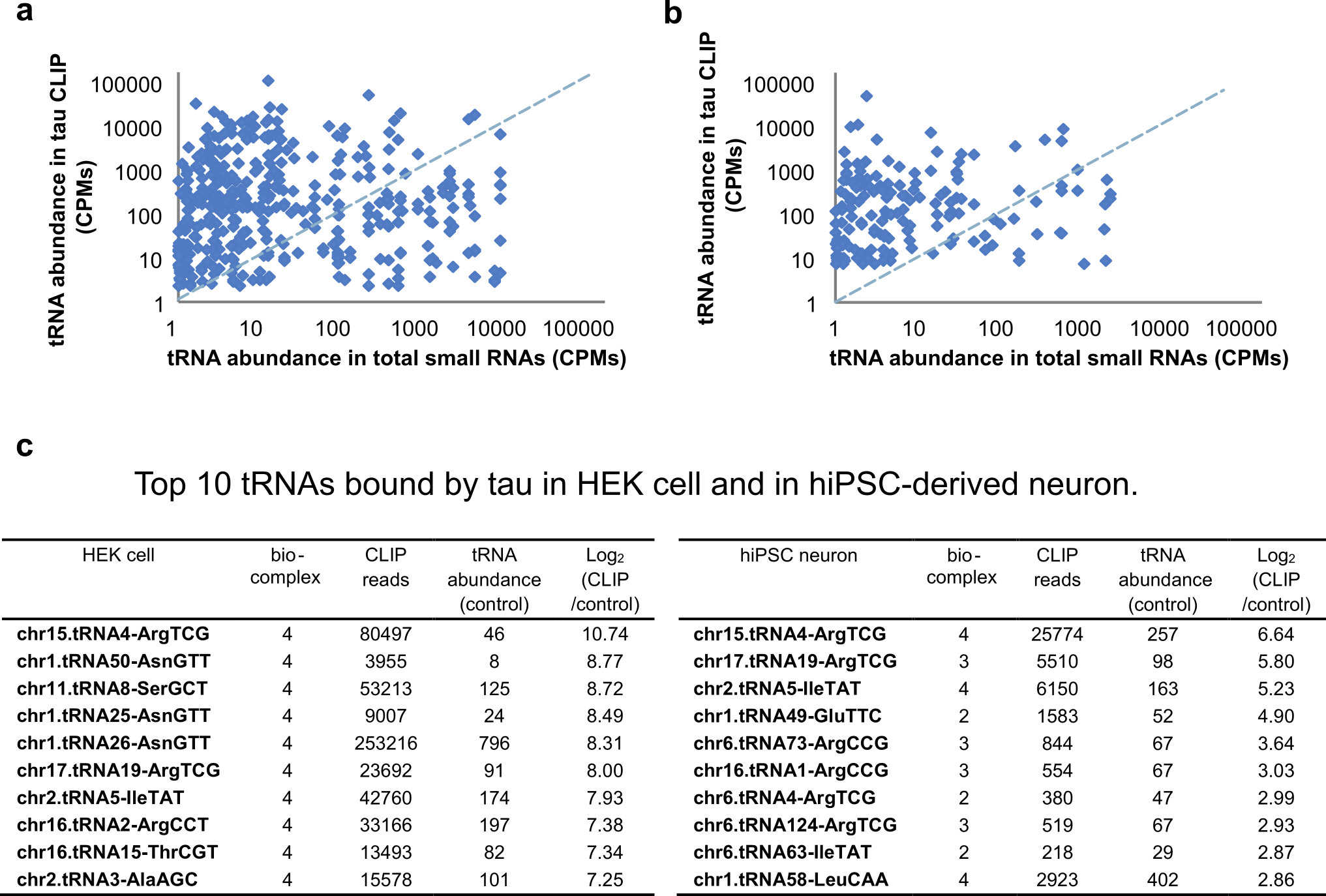

Supplement: S3 Fig — (a-b) tRNA abundance in CLIP samples vs total small RNA controls in HEK cells (a) and hiPSC-derived neurons (b) indicate that tRNA distributions differ between the total tRNA pool and the CLIP tRNA pool. Specifically, the population above the diagonal is much greater, indicating that the tRNA abundance from PAR-iCLIP samples from tau-bound RNA is significantly greater compared to the total small RNA present in HEK or hiPSC neuron cells. (c) Top 10 ranked tRNAs bound by tau in HEK cell and hiPSC-derived neurons. The numerical data used in a-b are included in S1 Data. (TIFF) [file pbio.2002183.s003.tiff]

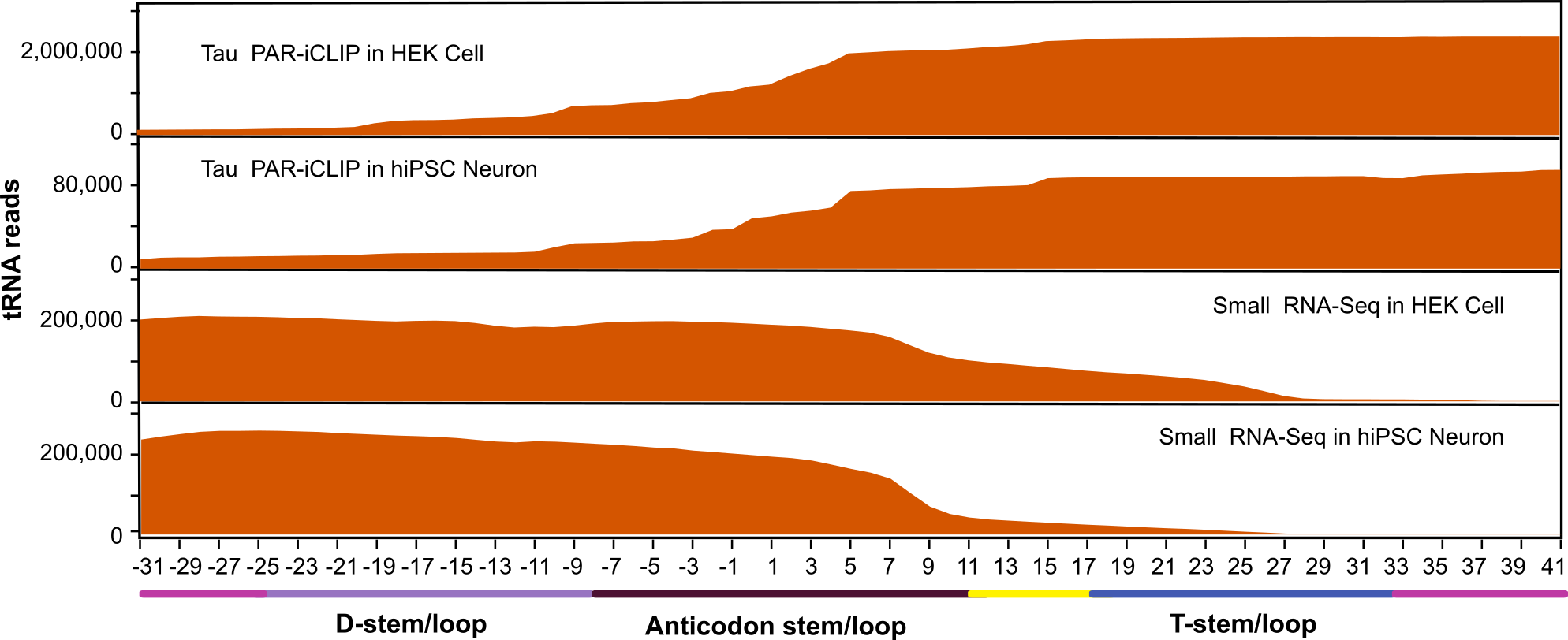

Supplement: S4 Fig — Accumulative CLIP cDNA reads from all tRNAs recovered in tau PAR-iCLIP of HEK cell and hiPSC-derived neurons (top two graphs) in comparison to the total small RNA population (small RNA-Seq) in HEK cells and hiPSC-derived neurons (bottom two graphs). The anticodon is designated as position 1–3 for alignment purpose, and the tRNA structure is shown below the x-axis in one dimension. Note the distinct pattern of crosslinking sites around the anticodon region in the PAR-iCLIP reads from both the Hek cells and hiPSC neurons in contrast to the expected 5’ bias of the tRNAs from the small RNA-Seq population. The numerical data used are included in S1 Data. (TIFF) [file pbio.2002183.s004.tiff]

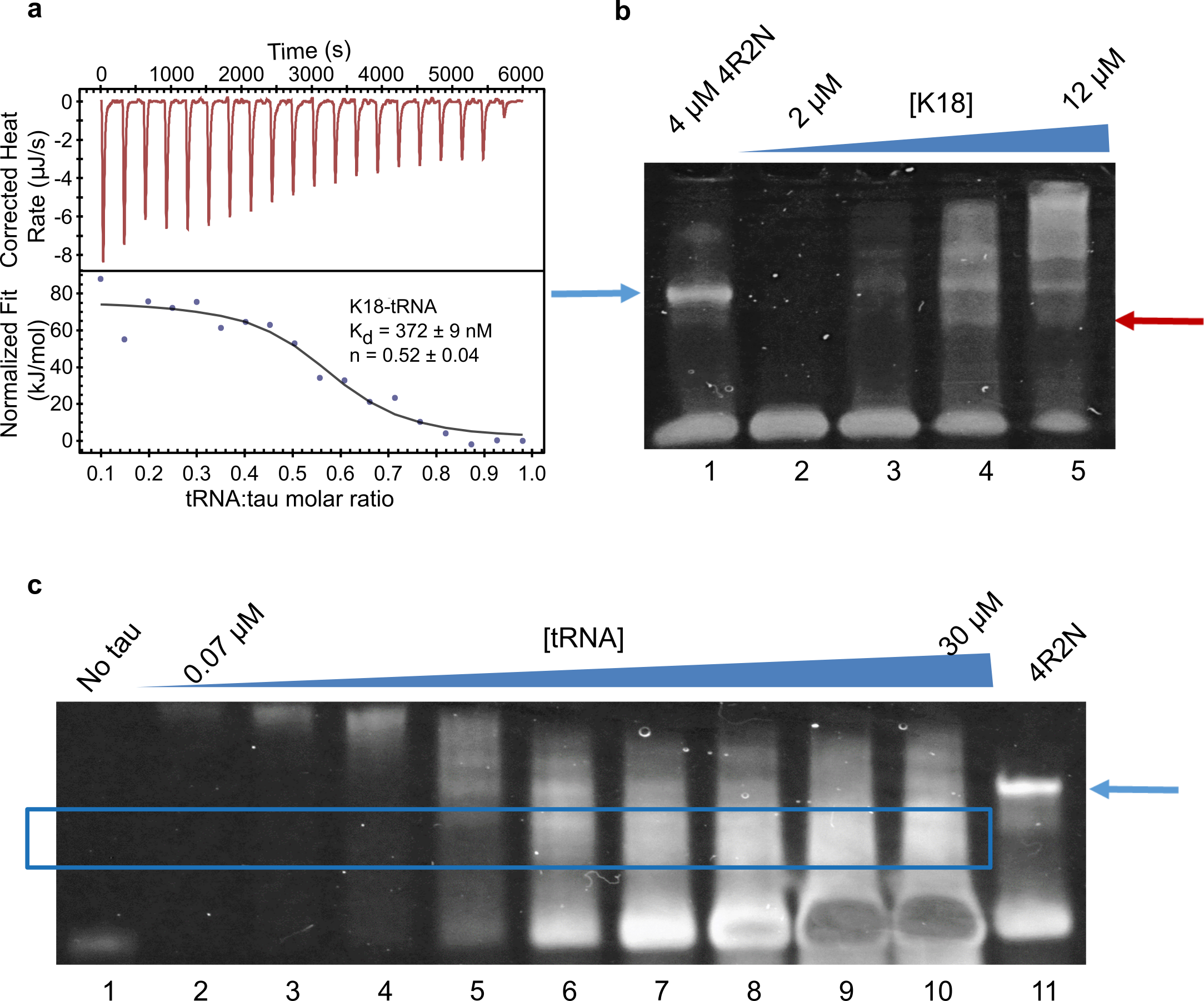

Supplement: S5 Fig — (a) Yeast tRNA was titrated into solutions of K18 tau in an ITC experiment. The top panels show the raw incremental-titration data. The area under each peak is integrated and plotted against the tRNA:tau molar ratio and fitted to an independent binding model (the bottom panel). The numerical data are included in S1 Data. (b) Tau-RNA binding experiments with varying tau:RNA molar ratio, while keeping tRNA concentration at 2.6 μM and changing the K18 concentration from 2 to 12 μM. Lane 1 shows 4 μM tau 4R2N as reference, and lane 2–5 2 μM, 4 μM, 6 μM, 12 μM of K18, respectively. The lower band of the 4R2N tau-tRNA or K18-tRNA complex are marked with blue and red arrows, respectively. The mobility of the lower band of the K18-tRNA complex is faster than that of the 4R2N tau-tRNA complex. (c) Tau-RNA binding experiments with varying tau:RNA molar ratio, while keeping tau concentration constant at 4 μM and incrementally increasing the tRNA concentration from 0.07 to 30 μM. Lane 1 shows the no-tau control with tRNA concentration at 0.07 μM, lane 2–10 shows 4 μM K18 with 0.07, 0.13, 0.26, 0.66, 1.3, 2.6, 6.6, 20, 30 μM tRNA, respectively; lane 11, and 4 μM tau 4R2N with 1.3 μM tRNA as reference. The highly concentrated RNA in lanes 8–10 hindered the penetration of the SYBR Gold II staining reagent. The lower bands of the K18-tRNA complexes are marked with a blue rectangle and of the 4R2N tau-tRNA complexes with a blue arrow. (TIFF) [file pbio.2002183.s005.tiff]

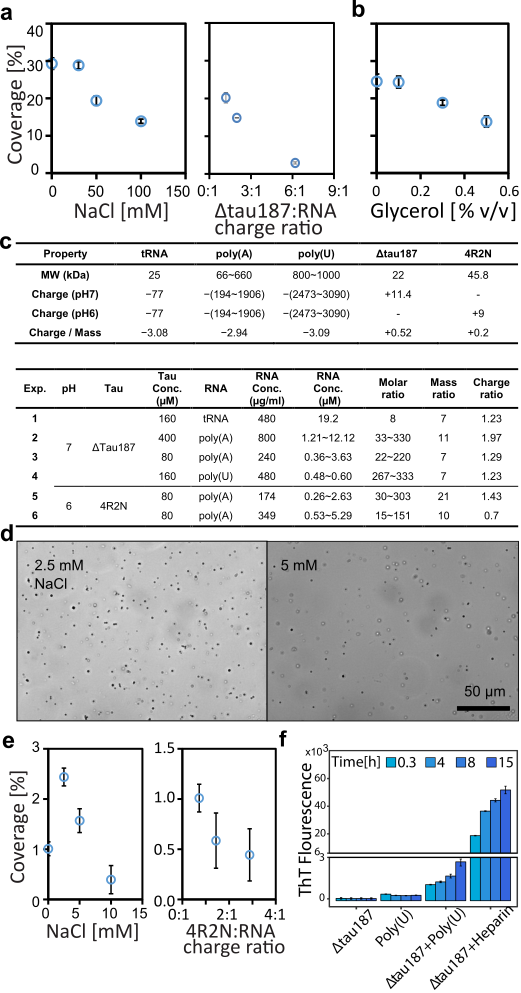

Supplement: S6 Fig — (a) Mixing Δtau187 with poly(U) RNA lead to droplet formation in vitro. Left: droplet coverage with varying NaCl concentration in samples made of 160 mM Δtau187 and 480 mg/ml poly(U). Right: droplet coverage by varying Δtau187:poly(U) charge ratios with no NaCl added. A total mass concentration of Δtau187 and poly(U) was kept at ~2 mg/ml, with 74 mM Δtau187 and 240 mg/ml poly(U) showing the highest droplet coverage (%). (b) Droplet coverage (%) of Δtau187 and poly(U) RNA with varying glycerol concentration. Experimental condition was maintained with 80 mM Δtau187 and 120 mg/ml poly(U), and with no added NaCl. (c) Calculations of tau-RNA molar or charge ratio, based on their chemical properties (top table), for various experimental conditions where droplet formation is observed (bottom table). The charge of the RNA and protein species at a given pH were estimated (following calculations discussed below in the S1 Text). (d-e) Mixing full length 4R2N tau with poly(A) RNA lead to droplet formation in vitro. (d) Representative bright-field images of 4R2N tau-RNA droplet at two NaCl concentrations. (e) Left: droplet coverage (%) with varying NaCl concentration, and at a charge ratio of tau 4R2N and RNA at 0.7:1. Right: droplet coverage with varying 4R2N tau:RNA charge ratios, with no NaCl added. A total polymer mass concentration ~3.6 mg/ml was maintained in the experiments in d-e, with 80 mM 4R2N and 349 mg/ml poly(A) showing the highest droplet coverage. All data and images in a-e were acquired 10 minutes after mixing of tau, RNA and 19% glycerol in a 20 mM ammonium acetate buffer at pH 7. (f) Extent of poly(U) induced fibrilization of Δtau187 measured by Thioflavin T assay compared to that of heparin of Δtau187. Pyrimidines can react with ThT and give strong background. For that reason, we used poly(U) to carry out this assay. Error bars show standard deviation from n = 3 in a-b, e-f and the numerical data used are included in S1 Data. (TIFF) [file pbio.2002183.s006.tiff]

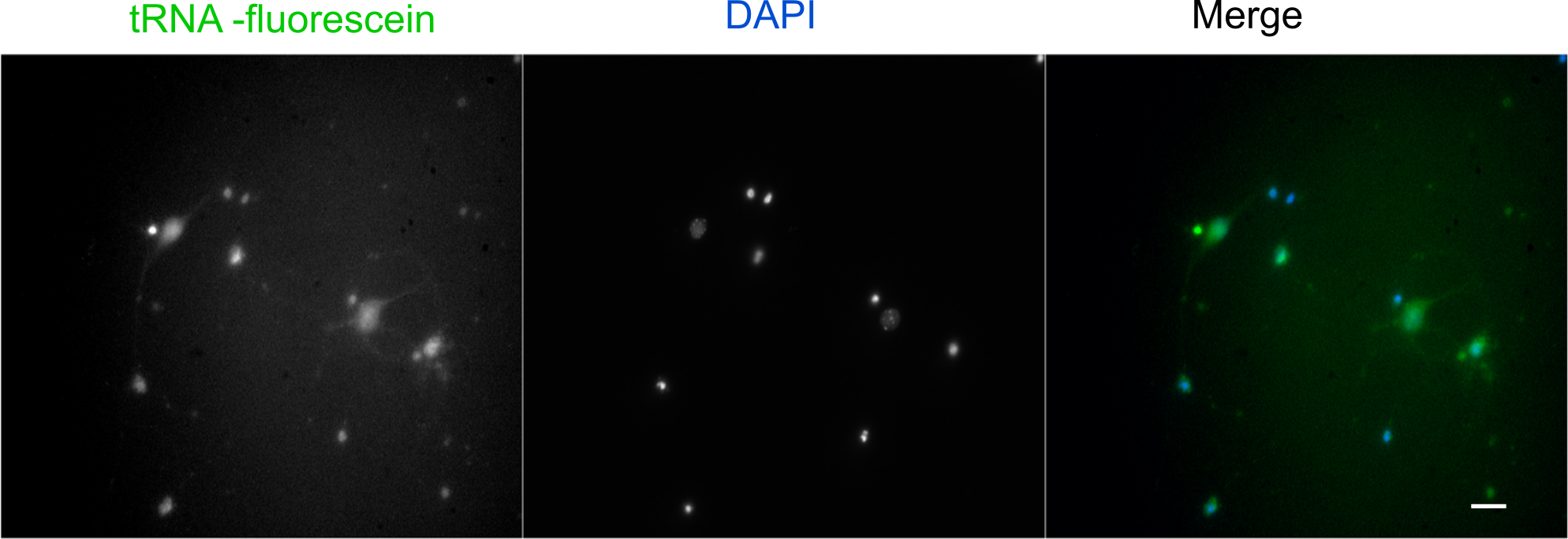

Supplement: S7 Fig — Primary mouse neurons at 14 days in vitro were transfected with 8 μg of tRNAPhe -fluorescein (green) per million cells using lipofectamine 2000. The images with tRNAPhe –fluorescein staining are shown together with DAPI (blue) staining of the nuclei. The tRNAPhe-fluorescein and DAPI-stained images show the colocalization of tRNA in the cell. Scale bar is 20 μm. (TIFF) [file pbio.2002183.s007.tiff]

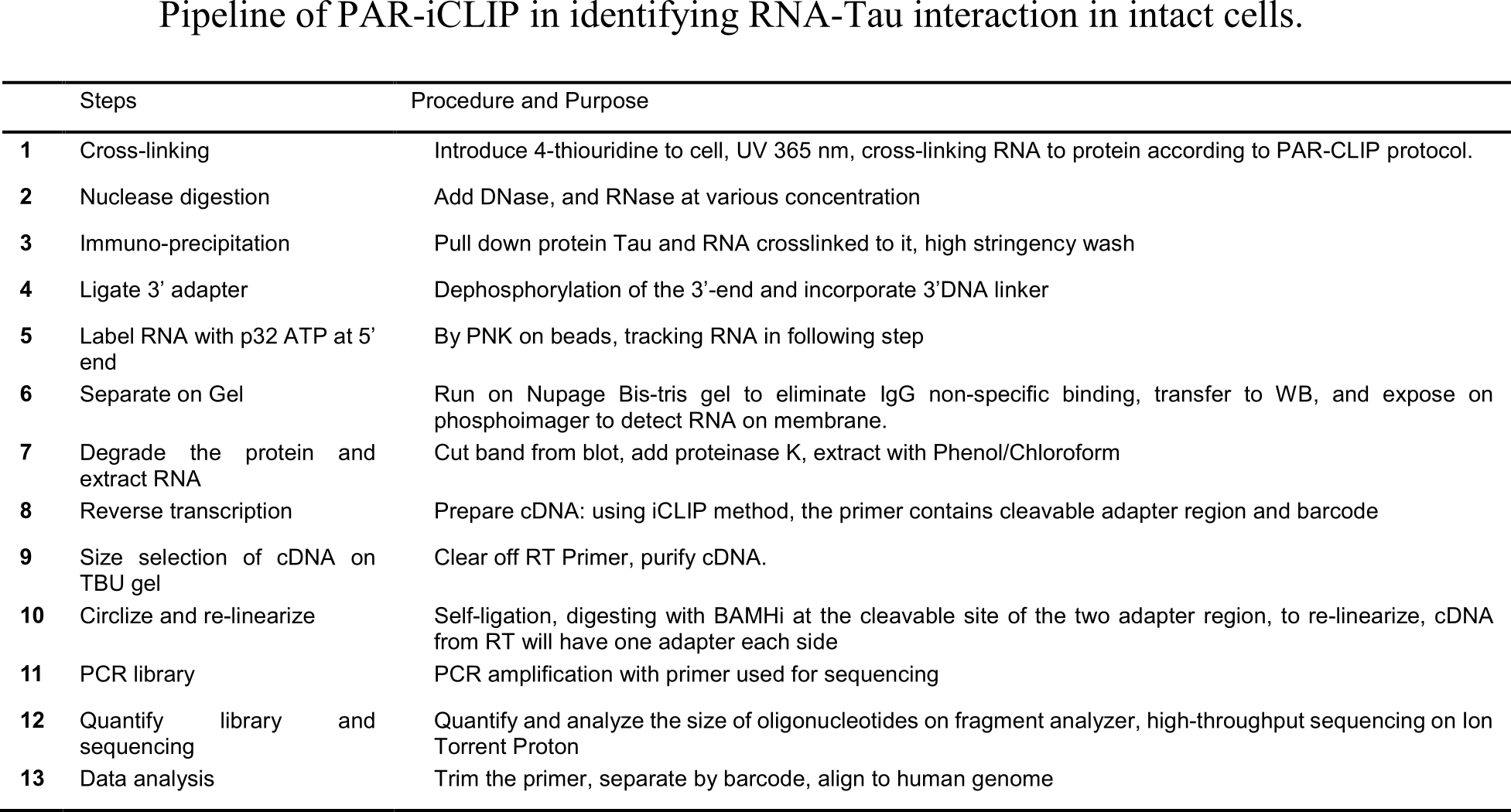

Supplement: S8 Fig — Pipeline of PAR-iCLIP in identifying RNA-tau interaction in intact cells. The protocol of PAR-iCLIP can be found in the Materials and Methods section of the manuscript, as well as in S1 Text. (TIFF) [file pbio.2002183.s008.tiff]
