# Supplementary material for: RNA stores tau reversibly in complex coacervates
Source: PLoS Biol. 2017 Jul 6;15(7):e2002183. doi: 10.1371/journal.pbio.2002183 (PMC5500003; doi:10.1371/journal.pbio.2002183)
Supplement: S1 Text — (DOCX) [file pbio.2002183.s009.docx]

**RNA Stores Tau Reversibly in Concentrated Coacervates**

S1 text

**Expression and purification of recombinant tau**

The gene of Δtau187, a truncated version of full length 4R2N tau which consists of residues 255-441, was cloned into the pET28a vector [1, 2]. The plasmid was then transformed and expressed using *E. coli* BL21 (DE3). Cell pellets were harvested and the resulting pellets were resuspended in lysis buffer (Tris-HCl pH 7.4, 100 mM NaCl, 0.5 mM DTT, 0.1 mM EDTA, 1mM PMSF) with 1 Pierce protease inhibitor tablet (Thermo Fisher). Lysis was initiated by the addition of lysozyme (2 mg/ml), DNase (20 µg/ml), and MgCl_2_ (10 mM) and incubated for 30 min on ice. Lysate was then sonicated for 10 min, boiled for 12 – 15 min. Samples were cooled on ice for 20 min and then centrifuged to remove the precipitant. The supernatant was loaded onto a Ni-NTA agarose column pre-equilibrated with wash buffer A (20 mM sodium phosphate pH 7.0, 500 mM NaCl, 10 mM imidazole, 100 µM EDTA). The column was then washed with 20 ml of buffer A, 15 ml buffer B (20 mM sodium phosphate pH 7.0, 1 M NaCl, 20 mM imidazole, 0.5 mM DTT, 100 µM EDTA), and then 10 ml of buffer A again. Purified Δtau187 was eluted with buffer C (20 mM sodium phosphate pH 7.0, 0.5 mM DTT, 100 mM NaCl) supplemented with varying amounts of imidazole increasing from 100 mM to 300 mM. The protein was then precipitated using an equal volume of methanol, in the presence of 10 mM DTT, at -20^o^C. The protein was then collected by centrifugation at 5,000 rpm at 4^o^C for 45 min. The final protein concentration was determined by UV-Vis absorption at 274 nm using an extinction coefficient of 2.8 cm^-1^mM^-1^, calculated from absorption of Tyrosine [3].

Full length tau protein (4R2N,1-441) or the microtubule binding domain (K18, 244-372) was purified with slight modification to previously published protocols [4]. Briefly, 4R2N tau in the pRK172 plasmid were expressed in E. coli BL21 (DE3). Cell pellets were harvested and resuspended in cell lysis buffer (50 mM MES pH 6.5, 5 mM DTT, 1mM PMSF, 1 mM EGTA) + complete protease inhibitor tablets (Roche). Lysate was sonicated, boiled for 10 min, and then centrifuged at 50,000 x g for 30 min at 4^o^C. The supernatant was then precipitated with ammonium sulfate (20% w/v) and centrifuged at 20,000 x g for 30 min at 4^o^C. The pellet was resuspended into 4ml of MonoS Buffer A (50 mM MES pH 6.5, 50 mM NaCl, 2mM DTT, 1 mM PMSF, 1mM EGTA) and dialyzed overnight against the same buffer. The protein was loaded on to a MonoS column (GE Healthcare) and eluted with a linear gradient of NaCl using MonoS Buffer B (Buffer A + 1 M NaCl). Fractions containing 4R2N tau were pooled, concentrated and dialyzed overnight into PBS pH 7.4. The final protein concentration was determined using a BCA assay (Thermo Fisher).

**Net charge of RNA**

To calculate the net charge of RNA, we assume one ribonucleotide monophosphate corresponding to one negative charge on the phosphate. Based on their pKa values, the four nuclear bases remain neutral in the range of pH 5-8. The one extra charge on each end of the RNA molecule can be ignored when the RNA contains more than 75 negative charges. Thus, the net charge is approximate to the numbers of bases in the chain. Also, the four ribonucleotide monophosphates have very similar molecular weights of around 321.5 ± 19.3. Even with mixed nuclear bases such as tRNA, the number of total ribonucleotide has a linear relationship to the weight of the RNA sample. Thus the net charge of the RNA sample can be relatively precisely determined using the total weight divided by the average MW of the monophosphate moiety.

Following this method, the calculation of the RNA charge is accurate, regardless of the length or type of the RNA used.

**Net charge of peptides**

The charge of a peptide at a given pH was estimated by applying an algorithm adapted from Innovagen’s Peptide Property Calculator (<http://pepcalc.com/>). All residues were assumed to have pKa values that are equivalent to the isolated residues (CRC Handbook of Chemistry and Physics, 87th ed). Charges of the C-terminus or N-terminus were estimated as -1 and +1, respectively at both pH 6.0 and pH 7.0. The processing code is made available on the internet (<https://github.com/yanxianUCSB/PeptideChargeCalculator)>.

**Charge ratio of tau and RNA**

Calculation of Charge ratio of tau and RNA is provided in the S1 Data.

**Detailed PAR-iCLIP protocol**

UV crosslinking

Cells were treated with 4-thiouridine (4SU) (Sigma-Aldrich) for 1h at a final concentration of 500 µM at 37°C, rinsed with ice-cold 1x PBS and irradiated once with UV light at 365 nm of 400 mJ/cm^2^ intensity on ice. The cells were centrifuged and the pellet stored at -80°C.

Bead preparation

Protein A/G plus beads (Thermo Fisher) (40 µl) were added to a spin column (Thermo Fisher) and washed 4 times with 500 µl of conjugation buffer (1x PSB made from 10x buffer using RNase-free water). Anti-tau antibody (HJ 8.5, 20 µl, 4.5 mg/ml) or anti-GFP (abcam, 1.5 µl) or anti-CDK5 (Santa Cruz, 40 µl, 0.2 mg/ml) was added to the beads in 800 µl conjugation buffer and the mixture was rotated on a bench top tube roller for 1 hour at room temperature. The supernatant was filtered and the beads were washed twice with 800 µl of conjugation buffer. The antibody was crosslinked to the beads using 800 µl of 450 µM DSS (Thermo Fisher) in conjugation buffer and rotated for 1 hour at room temperature. The supernatant was discarded and the reaction was quenched with 800 µl 0.2 M Tris buffer for 15 minutes at room temperature. The beads were then washed 3 times with 600 µl IP buffer (50 mM Tris pH 7.4, 150 mM NaCl, 1 % NP40) and resuspended in 0.8 ml lysis buffer (50 mM Tris pH 7.4, 150 mM NaCl, 1% NP40 and 1x protease inhibitor from Roche).

Cell lysis and partial RNA digestion

Lysis buffer (500 µl) was added to each cell pellet and incubated for 10 minutes on ice. For experiments using RNase, RNase I (Thermo Fisher) in lysis buffer were prepared, with a 1:50 dilution for high RNase and a 1:250 or 1:500 or 1:2000 dilution for low RNase concentration. 10 µl of diluted RNase was added to 500 µl of lysate. To save material, 2 µl of high RNase concentration was added to 100 µl lysate (1/5 total lysate) and used as reference for high RNase digestion. Turbo DNase (2 µl, Thermos Fisher) was then added to 500 µl lysate (0.4 µl Turbo DNase was added to 100 µl lysate). The samples were immediately placed at 37°C for exactly 3 minutes. Afterwards the samples were centrifuged at 20,000 x g for 10 minutes at 4°C. The supernatant was collected without touching the pellet. The protein concentrations of the supernatants were determined using the BCA protein assay kit (Thermo Fisher). The final concentration of protein was adjusted with lysis buffer to 0.4-1 mg in 500 µl. Input of the control was kept at the same amount for direct comparison.

Immunoprecipitation

Lysate (500 µl) was added to the beads and rotated overnight at 4°C. The next day the supernatant was discarded and the beads were washed twice with 600 µl cold high-salt buffer (50 mM Tris pH 7.4, 1 M NaCl, 1% NP-40, 1 mM EDTA), each for 5 minutes. The beads were then washed twice with 600 µl wash buffer (20 mM Tris pH 7.4, 10 mM MgCl_2_, 0.2% Tween‑20) and left in the last wash.

RNA 3’ end dephosphorylation

The supernatant was discarded and the beads were resuspended in 40 µl of the following solution mixture:

8 µl, 5x PNK pH 6.5 buffer (350 mM Tris, pH 6.5, 50 mM MgCl_2_, 5 mM DTT);

1 µl, PNK (NEB);

1 µl, RNasin (Promega);

30 µl, ddH_2_O.

The mixture was incubated and shaken at 1100 rpm for 20 minutes at 37°C, then washed once with 600 µl wash buffer and for at least 5 minute with 600 µl high salt buffer at 4°C. At the end it was washed twice with 600 µl of wash buffer.

L3 adapter ligation

The supernatant was discarded and the beads were resuspended in 40 µl of the following solution mixture:

4 µl, ddH_2_O;

10 µl, 4x ligation buffer (200 mM Tris-HCl pH 7.8; 40 mM MgCl_2_, 40mM DTT);

2 µl, T4 RNA ligase (NEB);

1 µl, RNasin (Promega);

3 µl, pre-adenylated adapter L3-App (20 µM)

(IDT rAppAGATCGGAAGAGCGGTTCAG/ddC/);

20 µl, PEG8000 50%.

The mixture was incubated at 16°C at 1100 rpm overnight. The next day 600 µl of wash buffer was added, then the mixture was washed twice with 600 µl of high salt buffer rotated at 4°C for 5 minutes and then washed twice with 600 µl of wash buffer.

5’ end labeling

Remove the supernatant and 36 µl of hot PNK mix was added to the beads:

1.8 µl, PNK (NEB);

3.6 µl, PNK 10x buffer (NEB);

29.4 µl, H_2_O;

1.2 µl, ATP [γ-^32^P].

The mixture was incubated at 37°C for 5 minutes. The radioactive solution was removed and discarded in the radioactive waste container.

Run 4-12% NuPAGE gel and transfer to nitrocellµlose membrane

2x NuPAGE gel-loading buffer with ddH_2_O was prepared and 25 µl was added to the beads. The samples were heated for 5 minutes at 70°C. The NuPAGE gel was run at 180 volts for 35-50 minutes with 20x MOPS-SDS running buffer (Thermo Fisher) and transferred for 90 minutes at 30 volt with 20x Transfer buffer (Thermo Fisher) to a nitrocellulose membrane (Protran *BA85*, VWR). The membrane was rinsed twice with 1x PBS and it was blotted briefly with Kimwipe at the back side. Then the membrane was wrapped with parafilm and placed on the top of the paper board, which has been unsymmetrically labeled with radioactive dye marker (prepared from blue marker dye and old ATP [γ-^32^P]) and wrapped in parafilm, for later positioning the radioactive signal. The packet of membrane and paper board was screened under phosphor imaging for 1 hour. The phosphor imager was scanned and analyzed using Quantity One software (Bio-Rad) and the membrane and paper board packet scanned for image alignment.

#### *RNA isolation*

An area of the nitrocellulose membrane that contained the target protein and up to an additional 45 kDa of macromolecules was cut out. The same region was cut out in the control sample as well as the region corresponding to the irrelevant protein GFP or CDK5. The membranes were transferred to a 1.5 ml tube, added to a mixture of 40 µL proteinase K (20 mg/ml, Thermo Fisher) in 160 µl PK buffer (100 mM Tris–HCl, pH 7.4, 50 mM NaCl, 10 mM EDTA), and shaken at 1100 rpm at 37°C for 30 min. Another solution containing 160 µl PK and 40 µl 20% SDS (final concentration of SDS is 2%) was subsequently added to the samples, and shaken at 1100 rpm at 55°C for 30 min. The supernatant together with 450 µl of saturated phenol: chloroform (1:1) mixture was added to a 2 ml Phase Lock Gel Heavy tube (VWR) and incubated for 5 min at 30°C, followed by centrifuging for 5 min at full speed at room temperature.

The aqueous layer was transferred into a new tube, and precipitated by adding 0.75 µL Glycoblue (Thermo Fisher) and 0.1 volume of 3 M sodium acetate pH 5.5 (Thermo Fisher), vortexing and adding 3x volume of 100% ethanol, mixed again and placed at -20°C overnight.

The mixture was centrifuged at 14,000 rpm at 4°C for 20 min. The radioactivity was monitored to ensure precipitation of RNA. The pellet was washed with 0.9 ml of 80% ethanol and spun again for 5 min, then resuspended in 5 µl of H_2_O and transferred to a PCR tube.

The further protocol was assumed from published procedures[5], except that for the PCR amplification the following primer mix specific to an Ion Torrent Proton (Thermo Fisher) DNA sequencer for one-directional sequencing (A-P5Solexa/trP1-P3Solexa) was used:

**A Target Forward P5Solexa**

5’-CCATCTCATCCCTGCGTGTCTCCGACTCAG|CACGACGCTCTTCCGATCT-3’ 49 nt

**trP1 Target Reverse P3Solexa**

5’-CCTCTCTATGGGCAGTCGGTGAT|CTGAACCGCTCTTCCGATCT-3’ 43 nt

**References:**

1. Peterson DW, Zhou H, Dahlquist FW, Lew J. A Soluble Oligomer of Tau Associated with Fiber Formation Analyzed by NMR. Biochemistry. 2008;47(28):7393-404. doi: 10.1021/bi702466a.

2. Pavlova A, Cheng CY, Kinnebrew M, Lew J, Dahlquist FW, Han S. Protein structural and surface water rearrangement constitute major events in the earliest aggregation stages of tau. Proc Natl Acad Sci U S A. 2016;113(2):E127-36. doi: 10.1073/pnas.1504415113. PubMed PMID: 26712030; PubMed Central PMCID: PMCPMC4720316.

3. Fasman GD, editor. Handbook of Biochemistry and Molecular Biology. 3rd ed. Cleveland, OH.: CRC Press; 1976.

4. Mulot SF, Hughes K, Woodgett JR, Anderton BH, Hanger DP. PHF-tau from Alzheimer's brain comprises four species on SDS-PAGE which can be mimicked by in vitro phosphorylation of human brain tau by glycogen synthase kinase-3 beta. FEBS Lett. 1994;349(3):359-64. PubMed PMID: 8050597.

5. Huppertz I, Attig J, D’Ambrogio A, Easton LE, Sibley CR, Sugimoto Y, et al. iCLIP: Protein–RNA interactions at nucleotide resolution. Methods. 2014;65(3):274-87. doi: <http://dx.doi.org/10.1016/j.ymeth.2013.10.011>.
